# Antimicrobial Use in Canadian Cow–Calf Herds

**DOI:** 10.3390/vetsci10050366

**Published:** 2023-05-20

**Authors:** Jayce D. Fossen, John R. Campbell, Sheryl P. Gow, Nathan Erickson, Cheryl L. Waldner

**Affiliations:** 1 Department of Large Animal Clinical Sciences, Western College of Veterinary Medicine, University of Saskatchewan, 52 Campus Drive, Saskatoon, SK S7N 5B4, Canada; 2 Public Health Agency of Canada, Saskatoon, SK S7N 5B4, Canada

**Keywords:** cow–calf, antimicrobial use (AMU), antimicrobial resistance (AMR), beef cattle

## Abstract

**Simple Summary:**

As of 1 December 2018, all medically important antimicrobials (MIA) for veterinary use in Canada required a prescription from a veterinarian for purchase. Cow–calf herds, while numerous and an important component of the food supply chain, have not been included in Canadian national on-farm antimicrobial-use surveillance. Studies of antimicrobial-use practices on extensively managed cow–calf operations are limited, and prior to this study, the effects of the regulatory changes on antimicrobials in cow–calf herds had not been reported. The objectives of this study were to describe antimicrobial use practices on cow–calf operations and any changes associated with the imposed regulations. Overall, antimicrobial use patterns in 2020 remain relatively unchanged in cow–calf herds after the regulatory changes and compared to previous comparable data from 2014. While almost all herds used antimicrobials, the frequency of antimicrobial use within cow–calf operations was low. Antimicrobials were primarily used for the treatment of respiratory disease in calves, neonatal diarrhea, and lameness in adults; however, most herds treated less than 5% of animals for these reasons. The most frequently reported antimicrobials, tetracyclines and florfenicol, were not classified as either high or very high importance to human health.

**Abstract:**

Despite growing concern surrounding antimicrobial use (AMU) and the importance of cow–calf herds to the Canadian livestock industry, surveillance of AMU in cow–calf herds to inform antimicrobial stewardship programs has been sporadic. Producers from the Canadian Cow–Calf Surveillance Network (87%, 146/168) provided data and almost all reported AMU in at least one animal (99%, 145/146 herds) in 2019–2020. The most common reasons for AMU were treatment of respiratory disease in nursing calves in 78% of herds and neonatal diarrhea in 67% of herds, as well as for lameness in cows in 83% of herds. However, most herds treated <5% of animals for these reasons. Less than 2.5% of herds treated more than 30% of calves for either bovine respiratory disease or neonatal diarrhea and no herds treated more than 30% of cows for lameness. The most frequently reported antimicrobial was oxytetracycline in 81% of herds, followed by florfenicol in 73% of herds. Antimicrobials with very high importance to human health, such as ceftiofur, were used at least once by 20% of herds but were only used in >30% of nursing calves from one herd. Similarly, while 56% of herds used macrolides at least once, within-herd use was the highest in nursing calves where <4% of herds reported use in >30% of animals. Herds using artificial insemination and calving in the winter were more likely (*p =* 0.05) to treat >5% of nursing calves for respiratory disease, suggesting the importance of vaccination programs for herds at risk. Overall, AMU was similar to previous Canadian studies; however, the percentage of herds using macrolides had increased from a comparable study in 2014.

## 1. Introduction

Antimicrobial use (AMU) and antimicrobial resistance (AMR) are sources of increasing concern for both human health and the livestock industry [1]. In Canada, two government-supported programs estimate livestock exposure to antimicrobials. The first initiative focuses on antimicrobial sales and includes livestock commodities important to the Canadian economy. From sales data, the Canadian Integrated Program for Antimicrobial Resistance Surveillance (CIPARS) and the Veterinary Drugs Directorate (VDD) consolidate information on the types of antimicrobial products, as well as the total volume distributed for sale stratified by livestock species [2].

The second stream of AMU surveillance collects on-farm data but is limited to broiler chickens, grower-finisher pigs, turkeys, dairy, and feedlot cattle. In this stream, AMU is collected from sentinel farms that are enrolled in CIPARS farm surveillance providing qualitative and quantitative information on AMU as well as the reasons for use. Even with cow–calf operations being the most numerous livestock operations in Canada, with approximately 54,000 operations across the country, and despite cow–calf operations involving the most farm families of any commodity [3], this sector is not currently a part of ongoing on-farm federal surveillance programs. These operations directly contribute to the food chain through the sale of cull cows and bulls and are the first step in the beef production process supplying backgrounding operations and feedlots.

The Canadian government implemented regulations to enhance veterinary supervision over AMU in 2004, to automatically categorized newly approved antimicrobials as medically important (MIA), thereby making them available by prescription only. In December 2018, new regulations required all MIA antimicrobials, including those approved before 2004, to have a veterinary prescription for purchase by producers [4]. Before the 2018 regulations for antimicrobial sales, the most recent Canadian report describing AMU from western Canadian cow–calf herds was in 2014 [5,6]. There are no recent data to assess the impact of the December 2018 regulatory change on the cow–calf industry.

There are also no recently published data on AMU in cow–calf herds located in Eastern Canada with the last report published in 2008 [7]. The lack of data limits any assessment of regulatory changes and other efforts to enhance antimicrobial stewardship in the Canadian beef industry. The current study along with the recent release of data from the 2017 USDA beef cow–calf survey [8,9] allows for a timely comparison of AMU practices across the relatively extensively managed North American cow–calf industry.

The primary objectives of this study were to determine the types of antimicrobials used on Canadian cow–calf operations and the reason for AMU in different animal groups (nursing calves, weaned calves, cows, and bulls), as well as any regional differences. The second objective was to compare these data to previously collected baseline data to determine if there have been any changes within the industry since the recent regulatory changes.

## 2. Materials and Methods

### 2.1. Study Design

This study was approved by the University of Saskatchewan’s Behavioural Research Ethics Board (Beh-REB#309).

A paper-based survey to assess AMU was developed based on an AMU survey tool administered to Western Canadian producers in 2014 [6]. The survey is included as a supplemental file (File S1: Antimicrobial use survey). Survey development and testing were previously described [6]. In addition to the survey, producers were provided with an updated antimicrobial-drug handbook (June 2019) to aid recall. The handbook included commercial and generic drug names with colour photographs of antimicrobial products approved for use in Canada.

### 2.2. Survey Content

The survey consisted of two sections. The first section collected AMU information between 1 July 2019, and 30 June 2020, by production group, namely nursing calves, weaned calves, breeding females, and bulls. Questions for each of the production groups were described in separate tables. They included whether any animals from the group had been administered antimicrobials for a list of specific reasons. The lists of production group-specific reasons included whether animals were given antimicrobials for disease prevention or treatment for diarrhea, umbilical infection, respiratory disease, lameness, eye disease, mammary disease, reproductive diseases, and others as appropriate for each production stage. If AMU was reported, the next questions addressed which antimicrobial product or products were administered for the specified reason, the percentage of animals in the herd which received each product (<5%, 6 to 30%, 31 to 70%, 71–100%), the typical number of doses used, dose per animal, and route of administration.

### 2.3. Participant Recruitment and Survey Distribution

In June 2020, hard-copy surveys were distributed to the 168 participants in the Canadian Cow–Calf Surveillance Network (C3SN) which was established in 2018 [10]. The C3SN included producers from all regions across Canada including 56 participants from its predecessor, the Western Canadian Cow–Calf Surveillance Network (WCCCSN) [5,6].

Herds were recruited through consultation with veterinarians, advertisements through research funding agencies such as the Beef Cattle Research Council (BCRC), provincial beef organizations, and word of mouth. Recruitment targeted herds larger than 40 breeding animals who reported pregnancy checking and had basic calving and production records. Network participants were provided with an honorarium for the completion of the surveys.

### 2.4. Data Management and Analysis

Using a commercial database program (Microsoft Access and Excel version 2304, Microsoft, Redmond, WA, USA) data from the 2020 AMU survey was merged with data from a 2019 general herd management, production, breeding, and weaning survey and data from a 2020 calving survey. These surveys were modified from a previous report [11]. Antimicrobial use was summarized for individual antimicrobials and classes of antimicrobials as a percentage of herds reporting use at least once for nursing calves, weaned calves, cows, and bulls, as well as based on the frequency of use the within herd and the reason for use. Differences in the proportion of herds using specific management practices and the relative frequency of AMU between herds from Western and Eastern Canada were compared using Fischer’s exact test.

Antimicrobial use was also summarized based on its importance to human health. Health Canada’s Veterinary Drugs Directorate, a division of the Government of Canada, developed a system for categorizing antimicrobials based on their importance to human health [12]: category I (very high importance, e.g., third-generation cephalosporins), category II (high importance, e.g., macrolides), category III (medium importance, e.g., tetracyclines), and category IV (low importance, e.g., ionophores). Categories I to III are considered medically important antimicrobials (MIA). Drugs of choice for treatment in human medicine are ranked higher as are antimicrobials where the availability of an alternative antimicrobial is limited.

Logistic regression was used to examine the associations between herd and producer attributes and responses to questions regarding the three diseases most treated with antimicrobials: respiratory disease in nursing calves, diarrhea in nursing calves, and lameness in cows. The outcome was whether the herd reported treating more than 5% of calves or cows for the condition of interest with at least one antimicrobial.

Exploratory models were constructed in STATA 16 (StataCorp, College Station, TX, USA) by first screening all unconditional associations that might be logically associated with the outcome to identify potential risk factors. Variables with a *p*-value < 0.20 were considered for inclusion in the final model. Multivariable models were created using manual backward selection. All factors with a *p <* 0.05 were retained in the final model and considered statistically significant. Potential confounders were retained in the model if inclusion changed the effect estimate by >25%. Two-way interactions were examined if more than two variables were retained as significant risk factors in the final model (*p <* 0.05) and the interaction was biologically plausible. Estimates were reported as odds ratios (OR) with 95% confidence intervals (95%CI). When intervening or highly correlated predictors were identified, competing models were assessed and the biologically appropriate model with the lowest Bayesian Information Criterion (BIC) was reported.

Risk factors explored included location of the herd (Western vs. Eastern Canada), herd size, number of animals purchased into the herd in 2020, whether a producer backgrounded calves, producer age, type of record-keeping system, season calving began, location of calving (pasture vs. any type of confinement system), use of community pasture, the use of breeding technologies including artificial insemination (AI), and herd type.

The number of animals purchased by each herd during the first half of the year was categorized as above or below the median for all herds and was considered a relative indicator of biosecurity risk for AMU for calf diarrhea and respiratory disease. For evaluating risk factors for AMU for cow lameness, herds were classified depending on whether they purchased more cattle than the median value for all herds throughout the year. Potential risk factors were selected based on previous publications [6,13] and expert opinion.

The secondary objective of the study was to compare the data collected in this publication to the previously collected baseline data to determine if there have been any changes within the industry since the imposition of the new regulations [4]. The relative frequency of specific outcomes of interest was compared between the 2020 and 2014 data [6] using Fischer’s exact tests.

## 3. Results

### 3.1. Description of Participating Herds

Returned surveys numbered 146 of 168 AMU, for a response rate of 87%: 30% of respondents were from Alberta, 20% from Saskatchewan, 17% from Ontario, 14% from Quebec, 12% from Manitoba, 5% from British Columbia, and 1% from both New Brunswick and Nova Scotia. Two-thirds of herds (67%, 98/146) were in Western Canada (British Columbia, Alberta, Saskatchewan, and Manitoba) and 33% (48/146) were in Eastern Canada (Ontario, Quebec, New Brunswick, and Nova Scotia). Out of the 56 who had previously participated in the WCCCSN [6], 52 returned a completed AMU survey.

The median number of cows calving per herd was 124 (range: 14 to 1044) and the median number of heifers calving was 18 (range: 0 to 210). The median number of cows calving per herd in Western Canada was 131 (range: 23 to 1044) and heifers calving was 19 (range: 0 to 210). Eastern herds were typically smaller with a median number of cows calving of 67 per herd (range: 14 to 578) and heifers of 8 (range: 0 to 109). The median number of bulls was 7 per herd (5th percentile, 2, 95th percentile 31).

For the 2019/2020 production cycle, most herd owners (79%, 115/146) reported maintaining individual animal treatment records (86%, 84/98 west; 65%, 31/48 east) (*p <* 0.001), 64% (94/146) reported herd-level records of total numbers of animals treated (67%, 66/98 west; 58%, 28/48 east) (*p =* 0.36), and 61% (89/146) reported maintaining both types of records (66%, 65/98 west; 50%, 24/48 east) (*p =* 0.07). Twenty-four herd owners reported using multiple systems for recording individual animal treatments, with most being handwritten (65%, 95/146). Smartphone systems were the second-most-popular option with 27 users (18%); 22 (15%) reported using a computer-based record system.

### 3.2. Overall Summary of AMU

Antimicrobial use data were collected for nursing calves, weaned calves, cows and bulls (Table 1, Table 2, Table 3 and Table 4). A greater proportion of producers reported treating adult cows and nursing calves at least once (Table 1, Table 3, Table A1 and Table A3) compared to weaned calves or bulls (Table 2, Table 4, Table A2 and Table A4). Almost all producers reported AMU at least once in a cow (95%, 139/146) or nursing calf (95%, 139/146). At least one cow was treated in 96% (94/98) of herds in the west and 92% (44/48) of herds in the east (*p =* 0.44). Similarly, 98% (96/98) of western herds reported AMU in nursing calves compared to 88% (42/48) in the east (*p =* 0.02). Antimicrobial use was reported at least once in weaned calves in 66% (96/146) of operations and for bulls in 65% (95/146) of herds; 71% (70/98) in the west and 54% (26/48) in the east for weaned calves (*p =* 0.04), and 74% (73/98) in the west and 46% (22/48) in the east for bulls (*p <* 0.001).

Medically important antimicrobials were used at least once in 99% (145/146) of herds within the study, with most herds using more than one category of antimicrobials (Table 5). Category I antimicrobials were used at least once by 33% (48/146) of herds, while category II products were used at least once by 80% (116/146) of herds, and category III by 94% (137/146) of herds. Use of category I antimicrobials at least once was similar in the west (36%, 35/98) and in the east (27%, 13/48) (*p =* 0.35). Category II antimicrobial use at least once was also not significantly different in the east (83%, 40/48) than in the west (78%, 76/98) (*p =* 0.62).

No MIAs or category I-III antimicrobials [12] were administered orally to adult cows or bulls (Table A7 and Table A8). All category IV antimicrobials were delivered orally (PO) across all animal classes. Category IV products (Table 6) were used by 14% of herds (21/146). More category IV use was reported in herds from Western Canada (17%, 17/98) than from Eastern Canada (8%, 4/48) (*p* = 0.21). Adult cows and weaned calves were most likely to receive in-feed category IV antimicrobials at least once. The proportion of animals within a herd treated with a category IV antimicrobial was not collected since this class of antimicrobial is provided in-feed for disease prevention and, therefore, typically given to all animals in a particular animal class.

Forty-five percent of herds reported the use of at least one MIA in less than 5% of animals in either nursing calves or cows (Table A15). Most herds (86%, 126/146) reported the use of MIA in less than 30% of nursing calves or cows. Twelve (8%) herds reported treatment of more than 70% of their nursing calves or cows with at least one MIA.

Ceftiofur was the most used category I injectable antimicrobial and was reported as being used at least once in 20% of herds as compared to danofloxacin (3%) and enrofloxacin (2%) (Table 5). Ceftiofur was reported as being used at least once in 23% (23/98) of herds in the west and 13% (6/48) in the east (*p =* 0.13) (Table A10).

Category I drug use [12] in more than 30% of nursing calves was reported in one (<1%) herd where ceftiofur was used to treat respiratory disease (Table 1, Table 2, Table 3 and Table 4). Category I drugs were not used in more than 30% of animals in weaned calves, cows, or bulls (Table 1, Table 2, Table 3 and Table 4).

Amongst all age categories, macrolides were the most likely to be used at least once of all the category II drugs (56% of the herds (81/146)) (Table 1, Table 2, Table 3, Table 4, Table 5, Table A9 and Table A10). Macrolides were used at least once in more herds from the west (61%, 60/98) than from the east (44%, 21/48) (*p =* 0.05) (Table A10).

Tetracyclines were the category III product most likely to be reported to be used at least once across all age categories (Table 5 and Table A10) and were used in 81% (118/146) of all herds (85%, 83/98 west; 73%, 35/48 east) (*p =* 0.09). Florfenicol was the second-most used at least once (Table 5) and was reported in 73% (107/146) of herds (84%, 82/98 west, 52%, 25/48 east) (*p <* 0.001). Sulfonamide boluses were the final product in this category and were used in 39% (57/146) of herds (Table 5); 40% (39/98) of herds in the west and 38% (18/48) of herds in the east (*p =* 0.79).

Of the 146 herds reporting, 4% (6/146) of herds reported the use of tetracyclines in more than 30% of nursing calves (Table 1 and Table A11). Tetracyclines were used by 3% (5/146) of herds in more than 30% of weaned calves (Table 2 and Table A12), and less than 1% (1/146) of herds in more than 30% of bulls (Table 4 and Table A14). No herds used tetracyclines in more than 30% of cows (Table 3 and Table A13). Florfenicol was used in greater than 30% of weaned calves in 1% (2/146) of herds, with less than 1% (1/146) of these herds using florfenicol in more than 70% of the weaned calves (Table 2). Florfenicol was not used in greater than 30% of nursing calves, cows, or bulls in any herd (Table 1, Table 3 and Table 4). Only 2% (3/146) of herds used sulfonamide boluses in greater than 30% of nursing calves (Table 1 and Table A11). No sulfonamide boluses were used in cows or bulls.

Lameness was the most common reason for the use of antimicrobials across all animal classes with 89% (130/146) of herds reporting that they treated for lameness at least once (Table 5). Respiratory infection was the second-most-common reason for treatment across all animal classes with 87% (127/146) of herds reporting treatment at least once (Table 5). The least-common reason for treatment across all animal classes was used for diseases that did not fall into the categories provided to producers and were identified as “other”. Only 5% (7/146) of herds reported “other” diseases, which included infection, injury, and dystocia (Table 5).

No producer reported using category I products for disease prevention (Table 5, Table A1, Table A2, Table A3 and Table A4). Category II products were used in 9% (13/146) of herds for disease prevention; macrolides in 5% (7/146) of herds and penicillins in 4% (6/146) of herds (Table A9). Category III products were used in 12% (17/146) of herds for disease prevention; tetracyclines in 10% (15/146) of herds and florfenicol in 1% (2/146) of herds (Table A9).

Products considered as MIA were used for the prevention of diseases such as diarrhea and pneumonia in 18% (26/146) of herds and all animal classes except for bulls (Table A1, Table A2, Table A3 and Table A4). AMU for disease prevention was highest for calves before weaning, with 12% (18/146) of herds reporting the use of MIA for disease prevention (Table A1); 15% (7/48) of herds from the east and 11% (11/98) of herds from the west used MIA for disease prevention (*p =* 0.60). For weaned calves, 5% (8/146) of herds (Table A2) and for cows, 1% (2/146) of herds (Table A3) reported using MIA for disease prevention.

No producer reported using more than two MIAs for disease prevention. The most common MIA used for disease prevention was oxytetracycline. Oxytetracycline was used for disease prevention in nursing calves, weaned calves, and cows in 8% (11/146), 2% (3/146), and 1% (2/146) of herds, respectively (Table A1, Table A2 and Table A3).

Sixty-two percent (90/146) of producers reported using remote delivery systems such as dart guns, crossbows, and pole syringes at least once to treat an animal. Remote delivery was more common in the west with 75% (73/98) of herds reporting use at least once compared to only 35% (17/48) of eastern herds reporting the use of a remote delivery system at least once. Dart guns were the most popular with 29% (42/146) of herds reporting use. Almost all dart gun use was in the west with 41% (41/98) of herds reporting dart gun use while only 2% (1/48) of herds used a dart gun in the east (*p <* 0.001). Most herds using dart guns (52%, 22/42) delivered less than 10% of total treatments using the dart gun. Nine producers reported using their dart gun to deliver greater than 50% of total treatments for sick animals.

A similar but smaller number of herds reported the use of crossbows (12%, 17/146) and pole syringes (8.9%, 13/146). Crossbows were used in 10% (10/98) of herds in the west and 15% (7/48) in the east (*p =* 0.43). Pole syringes were used in 11% (11/98) of western herds and 4% (2/48) of eastern herds (*p =* 0.22). Roping and restraining of sick cattle for treatment when handling facilities were not available was also reported by 25% (36/146) of herds; 28% (28/98) of herds in the west and 17% (8/48) of herds in the east (*p =* 0.15).

### 3.3. AMU in Nursing Calves

Ceftiofur, a category I antimicrobial, was used in nursing calves for the treatment of diarrhea in 8% (12/146) of herds and for respiratory infections in 6% (8/146) of herds (Table A1). Danofloxacin use was reported in 4% (6/146) of herds for treating respiratory infections in nursing calves (Table A1). Enrofloxacin use was reported in 2% (3/146) of herds; in one herd for treating nursing calves for respiratory disease, in one herd for diarrhea, and in one herd for navel infections (Table A1).

The category II drugs most likely to be used at least once in nursing calves were trimethoprim/sulfadoxine combinations, followed by penicillins and macrolides (Table 1 and Table A10). Trimethoprim/sulfadoxine combinations were most used to treat diarrhea, whereas penicillins were used for navel infections and arthritis (Table A1). Macrolides, most commonly tulathromycin and tilmicosin, were used for treating respiratory disease (Table A1).

The category III antimicrobials that were most likely to be used at least once in nursing calves were florfenicol followed by oxytetracycline (Table 1). Florfenicol was used primarily for respiratory disease, followed by navel infections (Table A1). Oxytetracycline was used for arthritis, followed by respiratory disease and navel infections (Table A1).

The number of herds reporting antimicrobial treatment for respiratory disease in calves before weaning varied by region (*p <* 0.001), with 86% (84/98) and 63% (30/48) of western and eastern herds, reporting this use, respectively. The number of herds reporting AMU for diarrhea was similar in Eastern and Western Canada (*p =* 0.99), with both 67% of eastern (32/48) and western (66/98) herds reporting AMU for diarrhea.

Respiratory infection was the most common reason for reporting AMU at least once in nursing calves (78%, 114/146) followed by diarrhea (67%, 98/146) (Table 5 and Table A1). Respiratory infection in nursing calves was most likely to be treated at least once with florfenicol or oxytetracycline. Trimethoprim/sulfadoxine followed by sulfonamide boluses were the most common antimicrobials used to treat diarrhea (Table A1).

Oxytetracyclines were the most common antimicrobials used for disease prevention in nursing calves (8%, 11/146) (Table A1). Benzylpenicillin procaine and benzathine penicillin were the second-most-reported MIA used for disease prevention in this age group, with 3% (4/146) of herds reporting use (Table A1). Two herds also reported using tulathromycin (1%) and one (<1%) herd reported the use of tildipirosin for disease prevention (Table A1).

The most common route of administration in nursing calves was subcutaneous (SQ) injection, followed by antimicrobial products being delivered orally (PO) (Table A5). Treatment of diarrhea via a bolus was the primary reason for oral AMU in nursing calves (43%, 64/146) (Table A1). The frequency of bolus use was similar across regions; 43% (43/98) in the west and 46% (21/48) in the east (*p =* 0.99).

### 3.4. AMU in Weaned Calves

AMU in weaned calves should be considered in the context that most herds weaned in October (38%, 55/146) or November (30%, 44/146). The median percentage of calves sold at weaning was 40%, and this did not differ between herds in the east (35%) and west (40%). Almost two-thirds of herds (64%, 93/146 all; 66%, 65/98 west; 58%, 28/48 east) also reported backgrounding some calves, and 14% (21/146 all; 9%, 9/98 west; 25%, 12/48 east) reported having a feedlot. Three herds did not retain any heifer calves as replacements.

Ceftiofur, a category I antimicrobial, was used for treating weaned calves with arthritis and eye (topical) infections in three herds (Table A2). Enrofloxacin use was reported in one herd for treating weaned calves for respiratory disease (Table A2). Penicillins were most commonly reported for the treatment of arthritis and eye infections (Table A2). Respiratory disease and arthritis were the main reason for the use of the category III antimicrobials tetracycline and florfenicol in weaned calves.

Similar to what was reported in nursing calves many of the farms using antimicrobials treated less than 5% of weaned calves (Table 2). Three percent (5/146) of herds reported treating more than 71% of weaned calves for various conditions including eye and respiratory disease with at least one category II or III product including tetracyclines, macrolides (gamithromycin, tulathromycin), and florfenicol.

Overall, respiratory disease was the most common reason for treatment in weaned calves (56%, 81/146) followed by treatment of arthritis (34%, 49/146) (Table A2). Florfenicol was the most reported drug for the treatment of respiratory disease, while oxytetracycline was the most frequent treatment for arthritis. Herd owners also reported treating weaned calves for eye disease. For disease prevention, the most reported conditions were pneumonia and diarrhea.

The most common method of administering antimicrobials to weaned calves was by SQ injections followed by intramuscular (IM) injections (Table A6), reflecting the drugs selected for use.

### 3.5. AMU in Breeding Females and Bulls

Parenteral ceftiofur was used to treat respiratory disease, reproductive disorders, and lameness in cows (Table A3). Similarly, bull lameness was the main reason for ceftiofur use (Table A4). Danofloxacin, another category I antimicrobial, was used for treating mastitis in <1% (1/146) of herds (Table A3); fluoroquinolones were not used for any other reason in cows nor were fluoroquinolones used in bulls for any reason. The only other category I drug reported was Polymyxin B which is a component of a commercial intramammary (IMM) preparation for mastitis. Fifteen percent of respondents (22/146) used this product at least once for mastitis. Producers also reported the topical use of intramammary preparations containing ceftiofur for treating eye infections in cows and bulls (Table A3 and Table A4).

Penicillins were the most common category II antimicrobials given to cows (Table 3 and Table A13). Penicillins were primarily used to treat lameness and reproductive-tract infections. However, the use of macrolides was also reported for treating lameness (<10% of herds) and reproductive tract infections (<3% of herds) in cows and bulls (Table A3 and Table A4). Macrolides were more likely to be used in cows from the west to treat lameness as compared to cows from the east (*p <* 0.001) (Table A10).

Oxytetracycline, a category III antimicrobial, was by far the most used antimicrobial in cows and bulls and was administered for a variety of reasons (Table A3 and Table A4). Florfenicol was also used to a lesser extent and was primarily administered for lameness. In addition to oxytetracycline, no additional MIAs were used for disease prevention in cows and no MIAs were used for disease prevention in bulls (Table A3 and Table A4).

No farms treated more than 30% of their cows; however, 3% (4/146) of farms treated over 30% of their bulls (Table 3 and Table 4); however, the median number of bulls in these herds was seven (5th percentile, 2, 95th percentile 31). Less than 20% of herds reported at least one treatment with an antimicrobial in more than 5% of cows (18%, 26/146) or bulls (13%, 19/146) (Table 3 and Table 4).

Lameness was the most common reason for AMU in adult cows and bulls (Table A3 and Table A4). Treatment of lameness was reported at least once by 83% (121/146) of herd owners for cows and 58% (84/146) for bulls. Treatment of lameness was reported at least once for cows by 87% (85/98) of herd owners in the west compared to 75% (36/48) in the east (*p =* 0.10). Treatment of lameness in bulls was reported in 68% (66/98) of western herds compared to 35% (16/48) of eastern herds (*p <* 0.001).

Lameness in adult animals was most frequently treated with oxytetracycline (Table A3 and Table A4). Oxytetracycline was also used for treating cows for eye and reproductive-tract infections (Table A3). Eye infection, often reported as pink eye, was the second-most-common reason for AMU in bulls followed by reproductive-tract and respiratory infections (Table A4).

The most popular route of administration in cows and bulls, as with weaned calves, was SQ injection followed by IM injection (Table A7 and Table A8).

### 3.6. Factors Associated with AMU

Antimicrobial use by antimicrobial class and percentage of the herd treated was summarized for the three most-common treatment scenarios: preweaning respiratory disease and diarrhea, as well as cow lameness (Table 7). The antimicrobial used by the greatest number of herds for the treatment of respiratory disease in nursing calves was florfenicol, an injectable phenicol. The antimicrobial reported as used by the greatest number of herds for the treatment of diarrhea in nursing calves was injectable trimethoprim sulfonamides and for cow lameness injectable tetracyclines (Table 7). Macrolides were the only antimicrobials used in >70% of animals within a herd for any of the three most-common diseases (Table 7). Macrolide use in >70% of calves was reported in one herd for the treatment and prevention of respiratory disease in nursing calves.

### 3.7. Comparison to 2014 Results

Twenty-one percent (30/146) of herds reported treating >5% of nursing calves with at least one antimicrobial for respiratory disease. Based on unconditional analysis (Table A16), herds calving earlier in the year and those herds using artificial insemination (AI) were more likely to treat >5% of nursing calves for respiratory disease. The use of breeding synchronization programs and having a record system also had *p <* 0.20 in the unconditional analysis. In the final multivariable model, which controlled for the calving season and the use of herd records, herds that used AI were more likely (OR 2.4, 95% CI: 1.0 to 5.9, *p =* 0.05) to treat >5% of their calves for respiratory disease compared to herds that did not use AI. Herds that calved in the winter (Dec to Feb) were also more likely (OR 2.5, 95% CI: 0.99 to 6.1, *p =* 0.05) to treat >5% of nursing calves for respiratory disease compared to herds that calved in the spring (March to May).

Diarrhea in nursing calves was the second-most-common reason for at least one calf to be treated prior to weaning (Table 7 and Table A1). However, calf diarrhea was the most common reason for treating at least 5% of the herd; 22% (32/146) of herds treated >5% of calves with at least one antimicrobial. Based on the unconditional analysis, no factors examined in this study were significantly associated with the treatment of >5% of nursing calves for diarrhea (Table A16). Only the calving season had a *p* < 0.20 in the unconditional analysis.

Lameness in adult cows was the most common reason for treatment at least once across all animal classes for all diseases in the study (Table 7 and Table A3); however, only 12% (18/146) of herds reported treatment of >5% of cows with an MIA (Table 7). Based on the unconditional analysis (Table A16) herds with a decision maker < 30 years old, those primarily seedstock producers, and those using community pastures were more likely to treat >5% of the herd (*p <* 0.20). In the final multivariable model, no factors were shown to lead to an increased likelihood of treating >5% of cows for lameness.

A summary table was developed to allow for comparison between this study from 2020, and a similar baseline study in 2014 (Table 8). The table contains the results from the 2020 study, which represents the entire country of Canada as well as the western-only data to allow for a more direct comparison with the 2014 study which only included Western Canada.

Overall, AMU was similar between the studies. However, there were some differences including an increase in macrolide use when comparing the 2020 western-only data to the 2014 data (61% vs. 44%) (*p =* 0.02) (Table 8). Macrolide use was also more often reported for treating respiratory disease in nursing calves in 2020 compared to 2014 (*p =* 0.03) (Table 8). The number of herds treating cows for lameness with a macrolide was nearly double for the 2020 study and higher for the western-only herds in the 2020 study versus the 2014 study (*p =* 0.002) (Table 8).

The use of some remote delivery systems also increased in western herds between 2014 and 2020. Use of dart guns in herds from the west increased from 18% (18/100) to 41% (41/98) (*p <* 0.001) and the use of pole syringes increased from 1% (1/100) to 11% (11/98) (*p =* 0.002).

## 4. Discussion

This is the first report of AMU data in Canadian cow–calf herds since 2013/2014 [5,6], the first since the new prescription-only regulations in 2018 [4], and the first to provide AMU data from cow–calf herds in Eastern Canada since 2008 [7]. The current study summarizes AMU from July 2019 to June 2020 in a sample of herds from across Canada. The current study provides information from Eastern Canada as well as from Western Canada. The more current data from Western Canada allows for discussion of any differences from the previous report [6] which also focused on a sample of Western Canadian cow–calf herds for the same period in 2013 and 2014.

Oxytetracycline and florfenicol were the most frequently reported antimicrobials used across all animal classes. These antimicrobials are considered of medium importance to human health (category III) by Health Canada [12]. Use of category III antimicrobials more frequently than categories of higher importance to human medicine in cow–calf herds supports antimicrobial stewardship by reducing selection pressure on antimicrobials of high and very high importance needed for treating more difficult infections.

Use of ceftiofur, a category I antimicrobial [12], was reported at least once in 20% of herds in at least one class of cattle, whereas category I fluoroquinolones were reported less frequently with 5% of herds reporting their use. Similar to the 2014 study [5,6], no herds reported the use of enrofloxacin or danofloxacin (injectable fluoroquinolones) in more than 5% of weaned calves or cows. Based on the most recent 2017 USDA beef study, the proportion of herds using injectable cephalosporins and fluoroquinolones is less than what was reported for Canada with only 1.6% and 0.9% of cow–calf herds in the USA reporting their use respectively [9]. However, in the 2017 USDA beef survey, producers were only asked to report their primary antibiotic, whereas, in the current study, they were asked to report up to three treatment options for each condition, making secondary drugs used in sicker animals or in the case of treatment failures more likely to be included. While in Canada, the proportion of animals for which category I antimicrobials are used within individual farms is relatively low, it would be prudent to carefully consider whether they are necessary for all herds where they are currently being used.

Trends in the primary diseases identified and the approaches to treatment in nursing beef calves have remained stable between the 2014 and 2020 studies. Respiratory disease and diarrhea were the two most-common reasons for using an antimicrobial at least once in nursing calves in both 2020 and 2014. While trends across the years were similar, there was a difference across the country in the 2020 data with the respiratory disease being treated more frequently in herds from Western Canada than from Eastern Canada. In the current study, 78% and 67% of herds treated at least one calf with an antimicrobial for respiratory disease or diarrhea, respectively, with a higher proportion of herds treating respiratory disease in the west than in the east. The relative importance of these diseases was similar to what has been recently observed in the American cow–calf industry, although the percentage of herds reporting treatment in the current study was higher. In the 2017 USDA beef study, 36% of medium-sized herds (50–199 cows) and 50% of larger herds (>200 cows) reported treating at least one calf for respiratory disease and 31% of medium-sized herds and 55% of larger herds for diarrhea [9].

Top treatment choices for respiratory disease and diarrhea did not change between 2014 [6] and 2020 with florfenicol most likely to be used for respiratory disease and oral sulfonamides followed by injectable sulfonamide–trimethoprim combinations most likely for diarrhea.

Macrolides are a category II drug considered of high importance to human health; however, macrolide use for treating respiratory disease in nursing calves was reported by a significantly higher proportion of herds in 2020 than in 2014. This change might be associated with the increasing availability of generic forms of one macrolide and the associated decrease in cost to producers, as well as the emergence of new macrolides. The use of macrolides for treating bovine respiratory disease (BRD) in nursing calves was also more common in 2020 in herds from the west than from the east.

Oral administration of MIAs was almost exclusively limited to the treatment of diarrhea in nursing calves via bolus administration. The proportion of herds using an oral MIA for the treatment of diarrhea in calves prior to weaning was 52% in 2014 [6] and 41% in 2020. There was, however, a significant decrease in the use of category II neomycin boluses from 11% in 2014 to 3% in 2020. In the 2017 USDA beef study, 11% of operations reported giving oral antimicrobials to nursing calves as the first-line treatment for diarrhea [9], which is much lower than what was seen either in the 2014 or the current study. The 2017 USDA beef study data were not stratified by herd size, and the study was dominated by small herds (<50 cows); however, the proportion of herds treating calves for scours was substantially higher in larger herds based on information from subsequent tables.

Lameness was the most reported reason for AMU in cows and bulls in 2020. Lameness was reported by relatively more western than eastern herds for both cows and bulls, with nearly double the herds treating bulls for lameness in the west as compared to the east. This difference is likely due to the relatively larger herd size and increased number of animals at risk in western herds. The 2017 USDA beef study [9] and the 2007–2008 USDA beef study [14] also found that lameness was the most-reported reason for treatment in adult cows. Lameness in adult cows was reported in 35% of medium-sized operations and 52% of large operations with approximately 80% of these herds reporting treatment with an antimicrobial [9]. The current study found that 83% of herds reported having and treating lame cows. This is considerably higher than the 35% and 52% reported by the medium- and large-sized herds in the USDA studies.

There are a number of reported differences in the proportion of herds treated for specific diseases between the American and Canadian studies. The reasons for the difference in the percentage of herds treated for respiratory disease, diarrhea, and lameness require further investigation. However, some possibilities could include climate differences in the primary cow/calf rearing areas, farm size, the season of calving, management intensity, recording keeping, and the approach to data collection and analysis.

The use of MIAs in 18% of herds for disease prevention (19% from the west) was similar in the present study to the 2014 study (21%) [6]. Disease prevention was most common in calves before weaning and was unchanged in 2020 with 12% (11% from the west) of herds compared to 11% in 2014 [5]. The most-common antimicrobial choices for preventative reasons also remained the same with oxytetracyclines and penicillins as the top choices. Unlike in 2014, no herds used category I products for disease prevention in 2020. While this finding could be due to additional veterinary oversight due to MIAs being prescription only, to definitively answer this question a targeted study would be needed.

In the previous 2014 study, 23% of cow–calf producers had reported the use of dart guns (18%), crossbows (10%), and pole syringes (1%) to treat on pasture where handling facilities were not available [6]. In the present study, the use of remote delivery systems was higher for the use of dart guns (31%) and pole syringes (9%). The use of crossbows (12%) was unchanged. Regardless of the remote delivery system used, less than 10% of treatments were delivered in this manner. Interestingly, there was a regional preference between Western and Eastern Canada with dart guns being significantly more commonly used in herds from the west. The 2017 USDA beef study [8] reported the use of pneumatic darts in 16% of medium-sized operations and 33% of large operations which was consistent with what was observed in Canada.

The main challenge with the use of remote delivery systems is that the total volume delivered by pneumatic darts is often less than 10 mL. This substantially limits the treatment options and typically results in the use of newer MIAs because often newer MIAs have a lower volume per unit of body weight dose [8]. Tulathromycin and tilmicosin were the two most-popular choices for pneumatic drug delivery in the 2017 USDA beef study [8]. The lower volume requirement might, in part, be why there has been an increase in the use of macrolides for treating lameness in cows. Lameness in cows is most common on summer pasture where access to handling facilities is limited and remote delivery tools can be a practical option for treatment.

While several herd attribute and management variables were examined, only a very small number were associated with the likelihood that more than 5% of animals treated for any of the three most-common reasons. The only factor significantly associated with the risk of treating more than 5% of nursing calves for respiratory disease in the final multivariable model was the use of AI. The reason for this association could be that, typically, calves are separated from their mother and comingled with other calves while producers process the cows, temporarily increasing animal stress and density, thereby potentially increasing the risk of pathogen transmission. This finding is consistent with previous reports from other studies [13,15,16] that identified the increasing number of times cow–calf pairs were gathered before turning out to summer pasture and estrus synchronization as risk factors for respiratory disease in calves before weaning.

Throughout the study, differences between Western and Eastern Canada were noted, including a higher frequency of western herds reporting AMU in nursing calves, weaned calves, and bulls. The variation in approaches to AMU could be due to differences in herd attributes and management practices. There were clear differences in herd size between eastern and western herds. Eastern herds reported a median of 75 bred cows and heifers whereas western herds had a median of 150 bred cows and heifers. Larger herds often require different management and could encounter different disease pressures than smaller herds. Herds from Western Canada were also more likely to maintain both individual-level records than herds located in Eastern Canada possibly due to less of an economic incentive for record maintenance in smaller operations [17]. Less record-keeping could mean more reliance on recall, potentially contributing to under-reporting.

Other studies have also reported differences between Western and Eastern Canadian cow–calf herds. In a recent summary of management practices, interventions near the time of birth were higher in eastern herds than in western herds [18]. Administration of intranasal respiratory vaccines and vitamins, and adequate colostrum intake were more common in Eastern Canada; however, overall herd vaccination levels were slightly lower in eastern herds [18]. While this recent BCRC report shows a greater uptake of these practices in Eastern Canada, a 2013 publication contains contradictory information and indicates that higher adoption of these practices was seen in Western Canadian herds, with the exclusion of overall vaccination levels for which similar data were reported [19].

A limitation of this study and all survey studies is recall bias. In this study, most producers reported maintaining herd (64%) and/or individual animal-treatment records (79%). The utilization of receipts and other sources of information was also encouraged to assist in the completion of the survey. However, historically, the quality/extent of records maintained by cow–calf operations has been questioned [7,17]. To address the issue of recall bias, measures were implemented in this study that were targeted to situations where records were not available or incomplete. For example, surveys were administered to producers in July 2020 immediately following calving season when most AMU is typically reported, the target time frame for the study, ensuring that the information if not recorded would still be relatively fresh in the producers’ minds. Additionally, producers were provided with an “antimicrobial handbook,” which included all licensed antimicrobials organized by route of administration, common names, generic names, and colour photographs of the product to aid recall regarding specific product use. Finally, when answers were unclear, participants were contacted by study personnel for clarification. Errors in reporting the choices of drugs used for particular diseases were expected to be relatively infrequent given the combined aids to recall, as were reports of the frequency of drug use. Rather than asking producers for specific treatment rates, producers were simply asked to report whether a specific product was used for a few animals (<5%), some animals (5–30%), many animals (30–70%), or most animals (>70%).

Social pressures including increased public scrutiny and stigmas in the beef industry around the use of antimicrobials in food production could have led to inaccurate data being provided by producers due to a perceived negative image or consequences related to what could be deemed “unnecessary use”. However, the impact of any hesitation to report use was expected to be minimal, given the need for a veterinary prescription for MIA, the commitment of the producers to completing surveys for this network over many years, the reporting of a range of antimicrobials, including very high importance category I products, and consistency of results across surveys, including surveys before AMU in the livestock industry was a target of public scrutiny.

Since the herds enrolled in the study were participants in a longitudinal surveillance network, the sample population is potentially representative of relatively more progressive, intensively managed herds than might be observed in a random sample. While the study gives insight into AMU practices on Canadian cow–calf operations, the data reflects a segment of Canadian herds of similar size under comparable management.

The percentage of herds and herd sizes enrolled from each province was, however, representative of all herds within Western and Eastern Canada. The herd sizes included in this study represent the cows from greater than 75% of all beef herds in Canada reported to the 2021 Agriculture Census [20]. Within Western Canada, the proportion of herds from each province was reflective of the 2021 Agriculture Census data reflecting the numbers of beef cows: Alberta 44%, Saskatchewan 30%, Manitoba 11%, and British Columbia 5% [20]. The proportion of herds from Eastern Canada in the present study was higher than that suggested by the distribution of beef cow–calf herds in Canada, Ontario 6%, Quebec 3%, and Maritimes 1% [20], to ensure there were sufficient numbers to provide information to regional producer groups.

Opportunities exist for improvement in the use of antimicrobials in cow–calf operations. The proportion of herds that used any antimicrobial at least once in more than 30% of either cows or nursing calves was relatively small at only 12%. By better understanding the reasons for use in these herds, it might be possible to target intervention strategies to address some of the primary reasons for use. The most apparent target for use reduction is the 12% of herds that reported the use of MIA for disease prevention in nursing calves.

## 5. Conclusions

The use of antimicrobials for the treatment of lameness was very common but is not necessary or beneficial in all cases. Noninfectious causes are generally responsible for most lameness in beef cattle [21]. For example, sand cracks are a known noninfectious cause of lameness in cattle. Studies have found that the prevalence of sand cracks in some Western Canadian herds resulted in up to 60% of the herd being affected [22]. Treatment of sand cracks with antimicrobials is typically not warranted.

Furthermore, the use of antimicrobials for the treatment of diarrhea in calves is another area where it might be possible to implement additional stewardship practices. Viruses can cause diarrhea in calves and viral causes of diarrhea will not respond to antimicrobials; however, it is challenging to differentiate viral and bacterial causes of diarrhea [23,24]. Veterinarians, producers, researchers, and the industry need to continue to work together to develop appropriate treatment guidelines and protocols and enhance biosecurity measures and vaccine uptake to help further reduce AMU in cow–calf herds. Some treatment guidelines and decision trees have been developed and are available to Canadian veterinarians through the Canadian Veterinary Medical Association [25] but at the time of this report, the decision tree was not yet available to producers. Future studies will be critical to inform stewardship protocols to meet the needs of this evolving industry.

In summary, there were few changes between 2014 and the 2020 AMU data presented in the current study. While there was little use of category I antimicrobials of very-high importance to human health in cow–calf herds, macrolide use did increase slightly between 2014 and 2020 in Western Canadian herds for respiratory disease in nursing calves and lameness in cows and bulls. Other regional differences were identified in the frequency of AMU for respiratory disease in nursing calves, the types of products used for treatment, and the use of remote delivery systems. The relative importance of different reasons for treatment is similar to the most recent surveillance report from the United States. However, the proportion of herds reporting use in the current study was higher than that for similar medium-sized herds in the US report.

## Figures and Tables

**Table 1:** Summary of antimicrobials used in nursing calves based on the percentage of herd treated.

Generic Antimicrobials for Nursing Calves	% Nursing Calves Receiving Antimicrobials Number (%) of Herds (n *=* 146) for Each Category					
	Herds with AMU (%) ^b^	<5%	6% to 30%	31% to 70%	71% to 100%	% Not Reported
Category I ^a^						
Ceftiofur	19 (13%)	17 (12%)	4 (2.7%)	1 (0.7%)		2 (1.4%)
Danofloxacin	4 (2.7%)	3 (2.1%)	2 (1.4%)			1 (0.7%)
Enrofloxacin	3 (2.1%)	2 (1.4%)	1 (0.7%)			
Penicillin G procaine/dihydrostreptomycin/ novobiocin/polymyxin B sulfate/ hydrocortisone acetate	2 (1.4%)	1 (0.7%)	1 (0.7%)			
Category II ^a^						
Ampicillin	2 (1.4%)	2 (1.4%)				
Benzylpenicillin procaine	30 (21%)	22 (15%)	6 (4.1%)		2 (1.4%)	1 (0.7%)
Benzylpenicillin procaine/benzathine	16 (11%)	10 (6.8%)	4 (2.7%)	1 (0.7%)	3 (2.1%)	1 (0.7%)
Gamithromycin	8 (5.5%)	8 (5.5%)	1 (0.7%)			1 (0.7%)
Neomycin sulfate/succinyl sulfathiazole	1 (0.7%)		1 (0.7%)			
Neomycin sulfate/sulfamethazine	4 (2.7%)	2 (1.4%)	2 (1.4%)			
Sulfadoxine/trimethoprim	46 (32%)	32 (22%)	13 (8.9%)	2 (1.4%)		2 (1.4%)
Sulfaguanidine/sulfathiazole/neomycin sulfate	1 (0.7%)		1 (0.7%)			
Tildipirosin	4 (2.7%)	3 (2.1%)		1 (0.7%)		
Tilmicosin	19 (13%)	16 (11%)	3 (2.1%)			1 (0.7%)
Tulathromycin	35 (24%)	24 (16%)	7 (4.8%)	3 (2.1%)	1 (0.7%)	2 (1.4%)
Category III ^a^						
Oxytetracycline	77 (53%)	65 (45%)	11 (7.5%)		6 (4.1%)	4 (2.7%)
Sulfaguanidine	18 (12%)	10 (6.8%)	7 (4.8%)			1 (0.7%)
Sulfamethazine	37 (25%)	23 (16%)	9 (6.2%)	2 (1.4%)		3 (2.1%)
Undefine bolus (Sulfonamide-based commercial product)	3 (2.1%)		1 (0.7%)	1 (0.7%)		1 (0.7%)
Total number of herds reporting use of any type of antimicrobial in nursing calves ^b^	138 (95%)	129 (88%)	59 (40%)	7 (4.8%)	12 (8.2%)	15 (10%)

^a^ Health Canada 2009, underlined product defines the category. ^b^ Rows and columns might not add up to the number of unique herds as herds can use one antimicrobial for different reasons at more than one frequency and more than one antimicrobial at the same frequency.

**Table 2:** Summary of antimicrobials used in calves postweaning.

Generic Antimicrobials for Weaned Calves	% Weaned Calves Receiving Antimicrobials in Each HerdNumber (%) of Herds (n *=* 146) for Each Category					
	Herds Reporting AMU (%) ^b^	<5%	6% to 30%	31% to 70%	71% to 100%	% Not Reported
Category I ^a^						
Ceftiofur	7 (4.8%)	7 (4.8%)	1 (0.7%)			
Enrofloxacin	1 (0.7%)	1 (0.7%)				
Penicillin G procaine/dihydrostreptomycin /novobiocin/polymyxin B sulfate/ hydrocortisone acetate—topical	3 (2.1%)	3 (2.1%)				
Category II ^a^						
Ampicillin	1 (0.7%)	1 (0.7%)				
Benzylpenicillin procaine	15 (10%)	14 (9.6%)	2 (1.4%)			
Benzylpenicillin procaine/benzathine	4 (2.7%)	2 (1.4%)	2 (1.4%)			
Gamithromycin	5 (3.4%)	4 (2.7%)			1 (0.7%)	
Sulfadoxine/trimethoprim	4 (2.7%)	3 (2.1%)	1 (0.7%)			
Tildipirosin	2 (1.4%)	2 (1.4%)				
Tilmicosin	17 (12%)	15 (10%)	2 (1.4%)			
Tulathromycin	21 (14%)	18 (12%)	2 (1.4%)		1 (0.7%)	1 (0.7%)
Category III ^a^						
Chlortetracycline hydrochloride	1 (0.7%)			1 (0.7%)		
Florfenicol	58 (40%)	46 (31.5%)	8 (5.5%)	1 (0.7%)	1 (0.7%)	2 (1.4%)
Oxytetracycline	57 (39%)	49 (34%)	9 (6.2%)	1 (0.7%)	4 (2.7%)	3 (2.1%)
Sulfamethazine	1 (0.7%)	1 (0.7%)				
Total herds reporting use of any type of antimicrobial in calves after weaning ^b^	96 (66%)	88 (60%)	23 (16%)	2 (1.4%)	5 (3.4%)	5 (3.4%)

^a^ Health Canada 2009, underlined product defines the category. ^b^ Rows and columns might not add up to the number of unique herds as herds can use an antimicrobial for different reasons at more than one frequency and more than one antimicrobial at the same frequency.

**Table 3:** Summary of antimicrobials used in cows based on the percentage of herd treated.

Generic Antimicrobials for Cows	%Cows Receiving Antimicrobials in Each Herd Number (%) of Herds (n *=* 146) for Each Category					
	Herds Reporting AMU (%) ^b^	<5%	6% to 30%	31% to 70%	71% to 100%	% Not Reported
Category I ^a^						
Ceftiofur	13 (8.9%)	12 (8.2%)	1 (0.7%)			1 (0.7%)
Danofloxacin	1 (0.7%)	1 (0.7%)				
Penicillin G procaine/dihydrostreptomycin/ novobiocin/polymyxin B sulfate/hydrocortisone acetate—IMM/topical	22 (15%)	19 (13%)	3 (2.1%)			
Category II ^a^						
Ampicillin	2 (1.4%)	2 (1.4%)				
Benzylpenicillin procaine	39 (27%)	37 (25%)	2 (1.4%)			2 (1.4%)
Benzylpenicillin procaine/benzathine	28 (19%)	25 (17%)	3 (2.1%)			2 (1.4%)
Cephapirin benzathine	3 (2.1%)	2 (1.4%)	1 (0.7%)			
Gamithromycin	7 (4.8%)	7 (4.8%)	1 (0.7%)			
Pirlimycin hydrochloride	1 (0.7%)	1 (0.7%)				
Sulfadoxine/trimethoprim	14 (9.6%)	12 (8.2%)				3 (2.1%)
Tildipirosin	3 (2.1%)	3 (2.1%)				
Tilmicosin	14 (9.6%)	14 (9.6%)				1 (0.7%)
Tulathromycin	15 (10%)	15 (10%)				2 (1.4%)
Category III ^a^						
Florfenicol	24 (16%)	22 (15%)	2 (1.4%)			
Oxytetracycline	104 (71%)	94 (64%)	19 (13%)			6 (4.1%)
Sulfanilamide	2 (1.4%)	1 (0.7%)				1 (0.7%)
Gramicidin (Not classified)	1 (0.7%)	1 (0.7%)				
Total herds reporting use of any type of antimicrobial in cows ^b^	138 (95%)	133 (91%)	26 (18%)	0 (0%)	0 (0%)	13 (8.9%)

^a^ Health Canada 2009, underlined product defines the category. ^b^ Rows and columns might not add up to the number of unique herds as herds can use an antimicrobial for different reasons at more than one frequency and more than one antimicrobial at the same frequency.

**Table 4:** Summary of antimicrobials used in bulls based on the percentage of herd treated.

Generic Antimicrobials for Bulls	% Bulls Receiving Antimicrobials for Each Herd Number of Herds (n = 146) (%) for Each Category					
	Herds Reporting AMU (%) ^b^	<5%	6% to 30%	31% to 70%	71% to 100%	% Not Reported
Category I ^a^						
Ceftiofur	4 (2.7%)	3 (2.1%)	1 (0.7%)			
Penicillin G procaine/ dihydrostreptomycin/ novobiocin/polymyxin B sulfate/ hydrocortisone acetate—topical	1 (0.7%)	1 (0.7%)				
Category II ^a^						
Ampicillin	2 (1.4%)	2 (1.4%)				
Benzylpenicillin procaine	9 (6.2%)	7 (4.8%)	1 (0.7%)			1 (0.7%)
Benzylpenicillin procaine/benzathine	14 (9.6%)	8 (5.5%)	3 (2.1%)	2 (1.4%)		1 (0.7%)
Gamithromycin	5 (3.4%)	4 (2.7%)				1 (0.7%)
Sulfadoxine/trimethoprim	2 (1.4%)		1 (0.7%)			1 (0.7%)
Tildipirosin	2 (1.4%)	2 (1.4%)				
Tilmicosin	13 (8.9%)	9 (6.2%)	2 (1.4%)	1 (0.7%)		2 (1.4%)
Tulathromycin	17 (12%)	16 (11%)				1 (0.7%)
Category III ^a^						
Florfenicol	9 (6.2%)	6 (4.1%)	1 (0.7%)			2 (1.4%)
Oxytetracycline	62 (43%)	48 (33%)	11 (7.5%)	1 (0.7%)		4 (2.7%)
Total herds reporting use of any type of antimicrobial in bulls ^b^	95 (65%)	77 (53%)	16 (11%)	4 (2.7%)	0 (0%)	9 (6.2%)

^a^ Health Canada 2009, underlined product defines the category. ^b^ Rows and columns might not add up to the number of unique herds as herds can use an antimicrobial for different reasons at more than one frequency and more than one antimicrobial at the same frequency.

**Table 5:** Summary of antimicrobials used and reasons for use in all animal classes from July 2019 to June 2020 in 146 cow–calf herds.

Generic Antimicrobials All Animal Classes	Total Number (%) Of Herds Reporting AMU										
	Use for ≥1 Reason	Arthritis	Diarrhea	Disease Prevention	Eye Infection	Lameness ^d^	Mastitis	Navel Infection	Other ^c^	Reproductive Tract	Respiratory Infection
Category i ^a^											
Ceftiofur	29 (20%)	2 (1.4%)	12 (8.2%)		4 (2.7%)	6 (4.1%)	4 (2.7%)	6 (4.1%)	1 (0.7%)	5 (3.4%)	8 (5.5%)
Danofloxacin	4 (2.7%)	2 (1.4%)	1 (0.7%)				1 (0.7%)	1 (0.7%)			4 (2.7%)
Enrofloxacin	3 (2.1%)		1 (0.7%)					1 (0.7%)			1 (0.7%)
Penicillin G procaine/dihydrostreptomycin / novobiocin/polymyxin b sulfate/hydrocortisone acetate	24 (17%)				5 (3.4%)		19 (13%)	1 (0.7%)	1 (0.7%)		
Category ii ^a^											
Ampicillin	2 (1.4%)	2 (1.4%)			1 (0.7%)	2 (1.4%)					
Benzylpenicillin procaine	54 (37%)	12 (8.2%)	2 (1.4%)	2 (1.4%)	10 (6.8%)	17 (12%)	7 (4.8%)	12 (8.2%)	5 (3.4%)	25 (17%)	5 (3.4%)
Benzylpenicillin procaine/ benzathine	35 (24%)	9 (6.2%)		4 (2.7%)	8 (5.5%)	25 (17%)	3 (2.1%)	5 (3.4%)	(0%)	8 (5.5%)	1 (0.7%)
Cephapirin benzathine	3 (2.1%)						1 (0.7%)			2 (1.4%)	
Gamithromycin	12 (8.2%)	3 (2.1%)	1 (0.7%)		3 (2.1%)	7 (4.8%)		1 (0.7%)			6 (4.1%)
Neomycin sulfate/ succinylsulfathiazole	1 (0.7%)		1 (0.7%)								
Neomycin sulfate/ Sulfamethazine	4 (2.7%)		4 (2.7%)								
Pirlimycin hydrochloride	1 (0.7%)						1 (0.7%)				
Sulfadoxine/trimethoprim	54 (37%)	2 (1.4%)	44 (30%)			2 (1.4%)	9 (6.2%)	5 (3.4%)	3 (2.1%)	3 (2.1%)	4 (2.7%)
Sulfaguanidine/sulfathiazole/ neomycin sulfate	1 (0.7%)		1 (0.7%)								
Tildipirosin	7 (4.8%)	2 (1.4%)	1 (0.7%)	1 (0.7%)		4 (2.7%)	1 (0.7%)				2 (1.4%)
Tilmicosin	32 (22%)	6 (4.1%)	2 (1.4%)	1 (0.7%)	2 (1.4%)	14 (9.6%)	4 (2.7%)	2 (1.4%)		2 (1.4%)	23 (16%)
Tulathromycin	43 (30%)	7 (4.8%)		5 (3.4%)	3 (2.1%)	19 (13%)	1 (0.7%)	5 (3.4%)		4 (2.7%)	34 (23%)
Category iii ^a^											
Chlortetracycline hydrochloride	1 (0.7%)				1 (0.7%)						
Florfenicol	107 (73%)	20 (14%)	16 (11%)	2 (1.4%)		10 (6.8%)	2 (1.4%)	46 (32%)	1 (0.7%)	1 (0.7%)	98 (67%)
Oxytetracycline	118 (81%)	45 (31%)	6 (4.1%)	15 (10%)	52 (36%)	96 (66%)	7 (4.8%)	17 (12%)	2 (1.4%)	37 (25%)	30 (21%)
Sulfaguanidine	18 (12%)		18 (12%)								
Sulfamethazine	37 (25%)	1 (0.7%)	36 (25%)								
Sulfanilamide	2 (1.4%)									2 (1.4%)	
Undefined bolus (sulfonamide-based commercial product)	3 (2.1%)		3 (2.1%)								
Gramicidin (not classified)	1 (0.7%)				1 (0.7%)						
Total number of herds reporting use of any antimicrobial ≥1 animal class ^b^	145 (99%)	82 (56%)	99 (68%)	26 (18%)	67 (46%)	130 (89%)	41 (28%)	88 (60%)	7 (4.8%)	69 (47%)	127 (87%)

^a^ Health Canada 2009, underlined product defines category. ^b^ Total does not reflect the sum of a column as some herds used more than one product for a particular reason. ^c^ Reasons for other treatment included broken bones, meningitis, improved feed efficiency, Caesarian sections, difficult calving, injuries, and infection. ^d^ Suspected infectious causes of lameness which may include footrot, arthritis, or digital dermatitis.

**Table 6:** Summary of Health Canada Category IV importance antimicrobial drug use (n *=* 146).

Generic Antimicrobial	Number (%) of Herds			
	Nursing Calves	Weaned Calves	Cows	Bulls
LASALOCID	1 (0.2%)		2 (1.4%)	2 (1.4%)
MONENSIN SODIUM	4 (2.7%)	11 (7.5%)	10 (6.8%)	3 (2.1%)
Total number of herds reporting use of Category IV antimicrobials ^a, b^	5 (3.4%)	11 (7.5%)	12 (8.2%)	5 (3.4%)

^a^ Health Canada 2009. ^b^ Total does not reflect the sum of a column as some herds used more than one product for a particular reason.

**Table 7:** Percentage of each herd treated with antimicrobials for the three most-common reasons for AMU.

Antimicrobial Category (Health Canada Category of Importance) ^a^	Total Herds Reporting AMU (%) ^b^	<5%	6 to 30%	31 to 70%	71 to 100%	% Not Reported
Nursing Respiratory Disease						
Injectable cephalosporin (3rd gen) (I)	8 (5.5%)	6 (4.1%)		1 (0.7%)		1 (0.7%)
Injectable fluoroquinolone (I)	5 (3.4%)	4 (2.7%)	1 (0.7%)			
Injectable macrolide (II)	48 (33%)	33 (23%)	8 (5.5%)	2 (1.4%)	1 (0.7%)	4 (2.7%)
Injectable penicillin (II)	5 (3.4%)	4 (2.7%)				1 (0.7%)
Injectable sulfonamide with trimethoprim (II)	3 (2.1%)	2 (1.4%)	1 (0.7%)			
Injectable phenicol (III)	87 (60%)	65 (45%)	19 (13%)			3 (2.1%)
Injectable tetracycline (III)	18 (12%)	14 (9.6%)	3 (2.1%)			1 (0.7%)
Any antimicrobial for respiratory disease in nursing calves ^b^	114 (78%)	94 (64%)	28 (19%)	2 (1.4%)	1 (0.7%)	7 (4.8%)
Nursing Diarrhea						
Injectable cephalosporin (3rd gen) (I)	12 (8.2%)	10 (6.8%)	2 (1.4%)			1 (0.7%)
Injectable fluoroquinolone (I)	2 (1.4%)	2 (1.4%)				
Injectable macrolide (II)	3 (2.1%)	1 (0.7%)	2 (1.4%)			
Injectable penicillin (II)	2 (1.4%)	1 (0.7%)	1 (0.7%)			
Injectable sulfonamide with trimethoprim (II)	42 (29%)	27 (19%)	12 (8.2%)	2 (1.4%)		1 (0.7%)
Injectable phenicol (III)	16 (11%)	12 (8.2%)	4 (2.7%)			
Injectable tetracycline (III)	4 (2.7%)	4 (2.7%)				4 (2.7%)
Oral antibiotic	61 (42%)	37 (25%)	19 (13%)	3 (2.1%)		
Any antimicrobial for diarrhea in nursing calves ^b^	98 (67%)	75 (51%)	30 (21%)	3 (2.1%)	0 (0%)	5 (3.4%)
Cow Lameness						
Injectable cephalosporin (3rd gen) (I)	3 (2.1%)	1 (0.7%)	1 (0.7%)			1 (0.7%)
Injectable macrolide (I)	30 (21%)	29 (20%)	1 (0.7%)			
Injectable penicillin (II)	34 (23%)	28 (19%)	4 (2.7%)			2 (1.4%)
Injectable sulfonamide with trimethoprim (II)	2 (1.4%)	1 (0.7%)				1 (0.7%)
Injectable phenicol (III)	7 (4.8%)	7 (4.8%)				
Injectable tetracycline (III)	84 (58%)	69 (47%)	13 (8.9%)			2 (1.4%)
Any antimicrobial for lameness ^b^	121 (83%)	108 (74%)	18 (12%)	0 (0%)	0 (0%)	4 (2.7%)

^a^ Health Canada 2009, underlined product defines the category. ^b^ Rows and columns might not add up to the number of unique herds as herds can use different antimicrobials within the same class at more than one frequency for the same reason or more than one class of antimicrobials for a specific reason at the same frequency.

**Table 8:** Data comparisons between 2020 and 2014 (baseline) data.

Outcome	2020 Data from All Herds (n *=* 146)	2020 Data from Western Canada (n *=* 98)	2014 Study from Western Canada (n *=* 100)	West Only 2020 Compared to 2014: *p*-Value
AMU at least once	99% (145/146)	100% (98/98)	98% (98/100)	0.50
Use of cephalosporin	20% (29/146)	24% (23/98)	15% (15/100)	0.15
Use of macrolides	55% (81/146)	61% (60/98)	44% (44/100)	0.02
AMU in Nursing Calves				
Respiratory Disease				
Herds treating nursing calves for respiratory disease	78% (114/146)	86% (84/98)	77% (77/100)	0.15
Herds treating more than 5% of nursing calves for respiratory disease	21% (30/146)	17% (17/98)	29% (29/100)	0.06
Use of cephalosporin for respiratory disease	6% (8/146)	6% (6/98)	3% (3/100)	0.33
Use of fluroquinolone for respiratory disease	3% (5/146)	2% (2/98)	4% (4/100)	0.68
Use of macrolides for respiratory disease	33% (48/146)	36% (35/98)	21% (21/100)	0.03
Diarrhea				
Herds treating nursing calves for diarrhea	67% (98/146)	67% (66/98)	73% (73/100)	0.44
Herds treating more than 5% of nursing calves for diarrhea	22% (32/146)	20% (20/98)	27% (27/100)	0.32
Use of cephalosporin for diarrhea	8% (12/146)	12% (12/98)	11% (11/100)	0.83
Use of fluroquinolone for diarrhea	1% (2/146)	1% (1/98)	1% (1/100)	0.99
Use of macrolides for diarrhea	2% (3/146)	3% (3/98)	1% (1/100)	0.37
Use of oral boluses for diarrhea	41% (61/146)	41% (40/98)	52% (52/100)	0.12
Use of neomycin boluses for diarrhea	3% (5/146)	2% (2/98)	11% (11/100)	0.02
AMU in Cows				
Herds treating cows for lameness	83% (121/146)	87% (85/98)	80% (80/100)	0.25
Herds treating more than 5% of cows for lameness	12% (18/146)	10% (10/98)	10% (10/100)	0.99
Use of cephalosporin for lameness in cows	2% (3/146)	3% (3/98)	0% (0/100)	0.12
Use of fluroquinolone for lameness in cows	0% (0/146)	0% (0/98)	0% (0/100)	0.99
Use of macrolides lameness in cows	21% (30/146)	29% (28/98)	11% (11/100)	0.002

## Data Availability

Data can not be shared except as in the aggregate form provided in the supplementary tables due to agreements with study participants.

## Supplementary Materials

The following supporting information can be downloaded at: https://www.mdpi.com/article/10.3390/vetsci10050366/s1, File S1: Antimicrobial use survey.

## Appendix A

**Table A1:** Summary of antimicrobials used and reasons for use in nursing calves from July 2019 to June 2020 in 146 cow–calf herds.

Generic Antimicrobials	Total Number of 146 Herds Reporting AMU in Calves Prior to Weaning							
	Use for ≥1 Reason	Arthritis	Diarrhea	Disease Prevention	Eye Infection	Navel Infections	Other ^c^	Respiratory Infection
Nursing calves								
Category I ^a^								
Ceftiofur	19 (13%)	2 (1.4%)	12 (8.2%)			6 (4.1%)		8 (5.5%)
Danofloxacin	4 (2.7%)	2 (1.4%)	1 (0.7%)			1 (0.7%)		4 (2.7%)
Enrofloxacin	3 (2.1%)		1 (0.7%)			1 (0.7%)		1 (0.7%)
Penicillin g procaine/dihydrostreptomycin/ novobiocin/polymyxin b sulfate/ hydrocortisone acetate	2 (1.4%)					1 (0.7%)	1 (0.7%)	
Category II ^a^								
Ampicillin	2 (1.4%)	2 (1.4%)						
Benzylpenicillin procaine	30 (21%)	12 (8.2%)	2 (1.4%)	2 (1.4%)		12 (8.2%)	2 (1.4%)	4 (2.7%)
Benzylpenicillin procaine/benzathine	16 (11%)	9 (6.2%)		4 (2.7%)	1 (0.7%)	5 (3.4%)		1 (0.7%)
Gamithromycin	8 (5.5%)	3 (2.1%)	1 (0.7%)		1 (0.7%)	1 (0.7%)	2 (1.4%)	4 (2.7%)
Neomycin sulfate/succinylsulfathiazole	1 (0.7%)		1 (0.7%)					
Neomycin sulfate/sulfamethazine	4 (2.7%)		4 (2.7%)					
Sulfadoxine/trimethoprim	46 (32%)	2 (1.4%)	44 (30%)			5 (3.4%)	1 (0.7%)	3 (2.1%)
Sulfaguanidine/sulfathiazole/neomycin sulfate	1 (0.7%)		1 (0.7%)					
Tildipirosin	4 (2.7%)	2 (1.4%)		1 (0.7%)				1 (0.7%)
Tilmicosin	19 (13%)	6 (4.1%)	2 (1.4%)		1 (0.7%)	2 (1.4%)		15 (10%)
Tulathromycin	35 (24%)	7 (4.8%)		2 (1.4%)	1 (0.7%)	5 (3.4%)	1 (0.7%)	29 (20%)
Category III ^a^								
Florfenicol	99 (68%)	20 (14%)	16 (11%)			46 (32%)	2 (1.4%)	87 (60%)
Oxytetracycline	77 (53%)	45 (31%)	5 (3.4%)	11 (7.5%)	8 (5.5%)	17 (12%)	5 (3.4%)	18 (12%)
Sulfaguanidine	18 (12%)		18 (12%)					
Sulfamethazine	37 (25%)	1 (0.7%)	36 (25%)					
Undefined bolus (sulfonamide-based commercial product)	3 (2.1%)		3 (2.1%)					
Total herds reporting use of any type of antimicrobial in calves before weaning ^b^	138 (95%)	82 (56%)	98 (67%)	18 (12%)	12 (8.2%)	89 (61%)	12 (8.2%)	114 (78%)

^a^ Health Canada 2009, underlined product defines the category. ^b^ Total does not reflect the sum of a column as some herds used more than one product for a particular reason. ^c^ Reasons for other treatment included infection, broken bones, and injuries from other animals including porcupine quills and predator attacks.

**Table A2:** Summary of antimicrobials used and reasons for use in weaned calves from July 2019 to June 2020 in 146 cow–calf herds.

Generic Antimicrobials	Total Number (%) of 146 Herds Reporting AMU in Calves after Weaning						
	Use for ≥1 Reason	Arthritis	Diarrhea	Disease Prevention	Eye Infection	Other ^c^	Respiratory Infection
Weaned Calves							
Category I ^a^							
Ceftiofur	7 (4.8%)	3 (2.1%)			3 (2.1%)	1 (0.7%)	2 (1.4%)
Enrofloxacin	1 (0.7%)						1 (0.7%)
Penicillin g procaine/ dihydrostreptomycin/novobiocin/ polymyxin b sulfate/hydrocortisone acetate—topical	3 (2.1%)				3 (2.1%)		
Sulfamethazine	1 (0.7%)		1 (0.7%)				
Category II ^a^							
Ampicillin	1 (0.7%)				1 (0.7%)		
Benzylpenicillin procaine	15 (10%)	9 (6.2%)			4 (2.7%)	1 (0.7%)	2 (1.4%)
Benzylpenicillin procaine/benzathine	4 (2.7%)	2 (1.4%)			3 (2.1%)		
Gamithromycin	5 (3.4%)	1 (0.7%)					4 (2.7%)
Oxytetracycline	57 (39%)	31 (21%)	1 (0.7%)	3 (2.1%)	25 (17%)	2 (1.4%)	14 (9.6%)
Sulfadoxine/trimethoprim	4 (2.7%)	1 (0.7%)	2 (1.4%)			1 (0.7%)	
Tildipirosin	2 (1.4%)		1 (0.7%)				2 (1.4%)
Tilmicosin	17 (12%)	3 (2.1%)		1 (0.7%)			14 (9.6%)
Tulathromycin	21 (14%)	6 (4.1%)		3 (2.1%)		1 (0.7%)	16 (11%)
Category III ^a^							
Chlortetracycline hydrochloride	1 (0.7%)				1 (0.7%)		
Florfenicol	58 (40%)	1 (0.7%)		2 (1.4%)		1 (0.7%)	57 (39%)
Total herds reporting use of any type of antimicrobial in calves after weaning ^b^	96 (66%)	49 (34%)	5 (3.4%)	8 (5.5%)	34 (23%)	5 (3.4%)	81 (56%)

^a^ Health Canada 2009, underlined product defines the category. ^b^ Total does not reflect the sum of a column as some herds used more than one product for a particular reason. ^c^ Reasons for other treatment included injuries, infection, improved feed efficiency, and meningitis.

**Table A3:** Summary of antimicrobials used and reasons for use in cows from July 2019 to June 2020 in 146 cow–calf herds.

Generic Antimicrobials	Total Number (%) of 146 Herds Reporting AMU in Cows							
	Use for ≥1 Reason	Disease Prevention	Eye Infection	Lameness ^d^	Mastitis	Other ^c^	Reproductive Tract	Respiratory Infection
Cows								
Category I ^a^								
Ceftiofur	13 (8.9%)		4 (2.7%)	3 (2.1%)	4 (2.7%)		5 (3.4%)	
Danofloxacin	1 (0.7%)				1 (0.7%)			
Penicillin g procaine/dihydrostreptomycin/novobiocin/polymyxin b sulfate/hydrocortisone acetate—IMM/topical	22 (15%)		4 (2.7%)		19 (13%)			
Category II ^a^								
Ampicillin	2 (1.4%)		1 (0.7%)	2 (1.4%)				
Benzylpenicillin procaine	39 (27%)		6 (4.1%)	14 (9.6%)	7 (4.8%)	2 (1.4%)	24 (16%)	1 (0.7%)
Benzylpenicillin procaine/benzathine	28 (19%)		6 (4.1%)	20 (14%)	3 (2.1%)	1 (0.7%)	8 (5.5%)	
Cephapirin benzathine	3 (2.1%)				1 (0.7%)		2 (1.4%)	
Gamithromycin	7 (4.8%)		3 (2.1%)	6 (4.1%)				1 (0.7%)
Pirlimycin hydrochloride	1 (0.7%)				1 (0.7%)			
Sulfadoxine/trimethoprim	14 (9.6%)			2 (1.4%)	9 (6.2%)	1 (0.7%)	3 (2.1%)	
Tildipirosin	3 (2.1%)			3 (2.1%)	1 (0.7%)			
Tilmicosin	14 (9.6%)		2 (1.4%)	8 (5.5%)	4 (2.7%)		1 (0.7%)	4 (2.7%)
Tulathromycin	15 (10%)		2 (1.4%)	14 (9.6%)	1 (0.7%)		1 (0.7%)	3 (2.1%)
Category III ^a^								
Florfenicol	24 (16%)			7 (4.8%)	2 (1.4%)			15 (10%)
Oxytetracycline	104 (71%)	2 (1.4%)	43 (30%)	84 (58%)	7 (4.8%)	3 (2.1%)	36 (25%)	7 (4.8%)
Sulfanilamide	2 (1.4%)						2 (1.4%)	
Gramicidin (Not classified)	1 (0.7%)		1 (0.7%)					
Total number of herds reporting use of any type of antimicrobial in cows ^b^	138 (95%)	2 (1.4%)	58 (40%)	121 (83%)	41 (28%)	5 (3.4%)	66 (45%)	25 (17%)

^a^ Health Canada 2009, underlined product defines the category. ^b^ Total does not reflect the sum of a column as some herds used more than one product for a particular reason. ^c^ Reasons for other treatment included Caesarean sections, assisted calvings, and pericarditis. ^d^ Suspected infectious causes of lameness which may include footrot, arthritis, or digital dermatitis.

**Table A4:** Summary of antimicrobials used and reasons for use in bulls from July 2019 to June 2020 in 146 cow–calf herds.

Generic Antimicrobials	Total Number (%) of 146 Herds Reporting AMU in Bulls					
	Use for ≥1 Reason	Eye Infection	Lameness ^d^	Other ^c^	Reproductive Tract	Respiratory Infection
Bulls						
Category I ^a^						
Ceftiofur	4 (2.7%)	1 (0.7%)	3 (2.1%)			
Penicillin g procaine/dihydrostreptomycin/novobiocin/polymyxin b sulfate/hydrocortisone acetate—topical	1 (0.7%)	1 (0.7%)				
Category II ^a^						
Ampicillin	2 (1.4%)		2 (1.4%)			
Benzylpenicillin procaine	9 (6.2%)	2 (1.4%)	3 (2.1%)	1 (0.7%)	2 (1.4%)	1 (0.7%)
Benzylpenicillin procaine/benzathine	14 (9.6%)	1 (0.7%)	12 (8.2%)	1 (0.7%)		
Gamithromycin	5 (3.4%)		5 (3.4%)			
Sulfadoxine/trimethoprim	2 (1.4%)		1 (0.7%)			1 (0.7%)
Tildipirosin	2 (1.4%)		2 (1.4%)			
Tilmicosin	13 (8.9%)	2 (1.4%)	11 (7.5%)		2 (1.4%)	1 (0.7%)
Tulathromycin	17 (12%)	2 (1.4%)	12 (8.2%)		4 (2.7%)	3 (2.1%)
Category III ^a^						
Florfenicol	9 (6.2%)		5 (3.4%)		1 (0.7%)	3 (2.1%)
Oxytetracycline	62 (43%)	10 (6.8%)	55 (38%)	3 (2.1%)	2 (1.4%)	3 (2.1%)
Total herds reporting use of any type of antimicrobial in bulls ^b^	95 (65%)	15 (10%)	84 (58%)	4 (2.7%)	11 (7.5%)	9 (6.2%)

^a^ Health Canada 2009, underlined product defines the category. ^b^ Total does not reflect the sum of a column as some herds used more than one product for a particular reason. ^c^ Reasons for other treatment included injury, infection or abscess, and improved feed efficiency. ^d^ Suspected infectious causes of lameness which may include footrot, arthritis, or digital dermatitis.

**Table A5:** Summary of medically important antimicrobials used and route of administration for nursing calves from July 2019 to June 2020 in 146 cow–calf herds.

Generic Antimicrobials	Site of Administration Prior to Weaning Number (%) of Herds (n *=* 146) for Each Category				
	Intravenous (IV)	Intramuscular (IM)	Subcutaneous (SQ)	SQ or IM	Orally (PO)
Nursing calves					
Category I ^a^ **					
Ceftiofur		2 (1.4%)	17 (11.6%)	1 (0.7%)	
Danofloxacin			4 (2.7%)		
Enrofloxacin		1 (0.7%)	2 (1.4%)		
Category II ^a^					
Ampicillin		2 (1.4%)			
Benzylpenicillin procaine		27 (19%)	1 (0.7%)	1 (0.7%)	1 (0.7%)
Benzylpenicillin procaine/benzathine		13 (8.9%)	3 (2.1%)		
Gamithromycin		2 (1.4%)	6 (4.1%)	1 (0.7%)	
Neomycin sulfate/succinylsulfathiazole					1 (0.7%)
Neomycin sulfate/sulfamethazine					4 (2.7%)
Sulfadoxine/trimethoprim	2 (1.4%)	41 (28%)	4 (2.7%)		
Sulfaguanidine/sulfathiazole/neomycin sulfate					1 (0.7%)
Tildipirosin			1 (0.7%)	3 (2.1%)	
Tilmicosin		2 (1.4%)	17 (12%)		
Tulathromycin		1 (0.7%)	33 (24%)		
Category III ^a^					
Florfenicol		17 (12%)	89 (61%)	2 (1.4%)	
Oxytetracycline		33 (23%)	33 (23%)	15 (10%)	
Sulfaguanidine					18 (12%)
Sulfamethazine					37 (25%)
Undefined bolus (sulfonamide-based commercial product)					3 (2.1%)
Total herds administering antimicrobials per site before weaning ^b^	2 (1.4%)	84 (58%)	115 (79%)	20 (14%)	60 (41%)

^a^ Health Canada 2009, underlined product defines the category. ^b^ Total does not reflect the sum of a column as some herds used more than one product for a particular reason. ** Penicillin g procaine/dihydrostreptomycin/novobiocin/polymyxin b sulfate/hydrocortisone acetate was administered topically in one calf and to an umbilical abscess in another.

**Table A6:** Summary of medically important antimicrobials used and route of administration for weaned calves from July 2019 to June 2020 in 146 cow–calf herds.

Generic Antimicrobials	Site of Administration Post Weaning Number (%) of Herds (n *=* 146) for Each Category				
	Intramuscular (IM)	Subcutaneous (SQ)	SQ or IM	Topical	Orally (PO)
Weaned Calves					
Category I ^a^					
Ceftiofur	1 (0.7%)	5 (3.4%)		1 (0.7%)	
Enrofloxacin		1 (0.7%)			
Penicillin g procaine/dihydrostreptomycin/novobiocin/polymyxin b sulfate/hydrocortisone acetate				3 (2.1%)	
Category II ^a^					
Ampicillin	1 (0.7%)				
Benzylpenicillin procaine	9 (6.2%)	4 (2.7%)	3 (2.1%)		
Benzylpenicillin procaine/benzathine	2 (1.4%)	3 (2.1%)			
Gamithromycin	2 (1.4%)	3 (2.1%)			
Sulfadoxine/trimethoprim	4 (2.7%)				
Tildipirosin		1 (0.7%)	1 (0.7%)		
Tilmicosin	1 (0.7%)	16 (11%)			
Tulathromycin	1 (0.7%)	20 (14%)			
Category III ^a^					
Chlortetracycline hydrochloride					1 (0.7%)
Florfenicol	6 (4.1%)	52 (36%)	1 (0.7%)		
Oxytetracycline	22 (15%)	31 (21%)	6 (4.1%)		
Sulfamethazine					1 (0.7%)
Total herds administering antimicrobials per site after weaning ^b^	40 (27%)	80 (55%)	11 (7.5%)	4 (2.7%)	2 (1.4%)

^a^ Health Canada 2009, underlined product defines the category. ^b^ Total does not reflect the sum of a column as some herds used more than one product for any reason.

**Table A7:** Summary of medically important antimicrobials used and route of administration for cows from July 2019 to June 2020 in 146 cow–calf herds.

Generic Antimicrobials	Site of Administration in Cows Number (%) of Herds (n *=* 146) for Each Category						
	Intravenous (IV)	Intramuscular (IM)	Subcutaneous (SQ)	SQ or IM	Intramammary (IMM)	Intrauterine (IU)	Topical
Cows							
Category I ^a^							
Ceftiofur		2 (1%)	6 (4.1%)		3 (2.1%)		2 (1.4%)
Danofloxacin			1 (0.7%)				
Penicillin g procaine/dihydrostreptomycin/novobiocin/polymyxin b sulfate/hydrocortisone acetate					18 (12%)	1 (0.7%)	4 (2.7%)
Category II ^a^							
Ampicillin		1 (0.7%)	1 (0.7%)				
Benzylpenicillin procaine		33 (23%)	2 (1.4%)	5 (3.4%)			
Benzylpenicillin procaine/benzathine		21 (14%)	7 (4.8%)	1 (0.7%)			
Cephapirin benzathine					1 (0.7%)	2 (1.4%)	
Gamithromycin		2 (1%)	5 (3.4%)				
Pirlimycin hydrochloride					1 (0.7%)		
Sulfadoxine/trimethoprim		12 (8.2%)	1 (0.7%)				
Tildipirosin			2 (1.4%)	1 (0.7%)			
Tilmicosin		1 (0.7%)	13 (8.9%)				
Tulathromycin		1 (0.7%)	15 (10%)				
Category III ^a^							
Florfenicol		7 (5%)	17 (12%)				
Oxytetracycline	1 (0.7%)	42 (29%)	46 (32%)	25 (17%)	1 (0.7%)	1 (0.7%)	
Sulfanilamide						2 (1.4%)	
Gramicidin (Not classified)							1 (0.7%)
Total herds administering antimicrobials per site in cows ^b^	1 (0.7%)	82 (56%)	71 (49%)	27 (19%)	23 (16%)	6 (4.1%)	6 (4.1%)

^a^ Health Canada 2009, underlined product defines the category. ^b^ Total does not reflect the sum of a column as some herds used more than one product for any reason.

**Table A8:** Summary of medically important antimicrobials used and route of administration for bulls from July 2019 to June 2020 in 146 cow–calf herds.

Generic Antimicrobials	Site of Administration in Bulls Number (%) of Herds (n *=* 146) for Each Category				
	Intravenous (IV)	Intramuscular (IM)	Subcutaneous (SQ)	SQ or IM	Topical
Bulls					
Category I ^a^					
Ceftiofur			3 (2.1%)		1 (0.7%)
Penicillin g procaine/dihydrostreptomycin/novobiocin/polymyxin b sulfate/hydrocortisone acetate					1 (0.7%)
Category II ^a^					
Ampicillin		1 (0.7%)	1 (0.7%)		
Benzylpenicillin procaine		6 (4.1%)	11 (0.7%)	2 (1.4%)	
Benzylpenicillin procaine/benzathine		12 (8.2%)	2 (1.4%)		
Gamithromycin		1 (0.7%)	4 (2.7%)		
Sulfadoxine/trimethoprim		1 (0.7%)	1 (0.7%)		
Tildipirosin		2 (1.4%)			
Tilmicosin		1 (0.7%)	12 (8.2%)		
Tulathromycin		3 (2.1%)	15 (10%)		
Category III ^a^					
Florfenicol		2 (1.4%)	7 (4.8%)		
Oxytetracycline	1 (0.7%)	23 (16%)	31 (21%)	10 (6.8%)	
Total herds administering antimicrobials per site in bulls ^b^	1 (0.7%)	45 (31%)	59 (40%)	12 (8.2%)	2 (1.4%)

^a^ Health Canada 2009, underlined product defines the category. ^b^ Total does not reflect the sum of a column as some herds used more than one product for any reason.

**Table A9:** Summary of antimicrobials used by antimicrobial class and reasons for use in all animal classes from July 2019 to June 2020 in 146 cow–calf herds.

Antimicrobial Class	Total Number of 146 Herds Reporting AMU										
	Use for ≥1 Reason	Arthritis	Diarrhea	Disease Prevention	Eye Disease	Lameness ^d^	Mastitis	Navel Infections	Other ^b^	Reproductive Tract Infections	Respiratory Infection
Category I ^c^											
Cephalosporins 3rd Gen	29 (20%)	2 (1%)	12 (8%)		4 (3%)	6 (4%)	4 (3%)	6 (4%)	1 (1%)	5 (3%)	8 (5%)
Penicillins/polymyxins	24 (16%)				5 (3%)		1 (1%)	1 (1%)	1 (1%)		
Polypeptides	1 (1%)				1 (1%)						
Quinolones and fluoroquinolones	7 (5%)	2 (1%)	2 (1%)				1 (1%)	2 (1%)			5 (3%)
Category II ^c^											
Aminoglycosides	5 (3%)		5 (3%)								
Cephalosporins 1^st^ Gen	3 (2%)						1 (1%)			2 (1%)	
Lincosamides	1 (1%)						1 (1%)				
Macrolides	81 (55%)	18 (12%)	4 (3%)	7 (5%)	7 (5%)	42 (29%)	6 (4%)	7 (5%)		6 (4%)	62 (42%)
Penicillins	74 (51%)	22 (15%)	2 (1%)	6 (4%)	18 (12%)	38 (26%)	10 (7%)	17 (12%)	4 (3%)	32 (22%)	6 (4%)
Sulfonamides with trimethoprim (injectable)	54 (37%)	2 (1.4%)	44 (30 %)			2 (1.4%)	9 (6.2%)	5 (3.4%)	3 (2.1%)	3 (2.1%)	4 (2.7%)
Category III ^c^											
Amphenicols	107 (73%)	20 (14%)	16 (11%)	2 (1%)		10 (7%)	2 (1%)	46 (32%)	1 (1%)	1 (1%)	98 (67%)
Sulfonamides (oral)	57 (39%)	1 (0.7%)	55 (38%)							2 (1.4%)	
Tetracyclines	118 (81%)	45 (31%)	6 (4%)	15 (10%)	52 (36%)	96 (66%)	7 (5%)	17 (12%)	2 (1%)	37 (25%)	30 (21%)
Total herds reporting use ^a^	145 (99%)	82 (56%)	99 (68%)	26 (18%)	67 (46%)	130 (89%)	41 (28%)	88 (60%)	7 (5%)	69 (47%)	127 (87%)

^a^ Total does not reflect the sum of a column as some herds used more than one product for a particular reason, ^b^ Reasons for other treatment included broken bones, meningitis, improved feed efficiency, Caesarian sections, difficult calving, injuries, and infection. ^c^ Health Canada 2009, underlined product defines the category. ^d^ Suspected infectious causes of lameness which may include footrot, arthritis, or digital dermatitis.

**Table A10:** Summary of antimicrobials used by antimicrobial class and animal class from July 2019 to June 2020 in 146 cow–calf herds.

Antimicrobial Class	Total Number of 146 Herds Reporting AMU					Western Canada: 98 Herds Reporting AMU					Eastern Canada: 48 Herds Reporting AMU					West vs. East All Use			
	All	Nursing	Weaned	Cows	Bulls	All	Nursing	Weaned	Cows	Bulls	All	Nursing	Weaned	Cows	Bulls	Odds Ratio	L 95% CI	U 95% CI	*p*-Value
Category I ^a^																			
Cephalosporins (3rd gen)	29 (20%)	19 (13%)	7 (4.8%)	13 (8.9%)	4 (2.7%)	23 (24%)	17 (17%)	4 (4.1%)	10 (10%)	3 (3.1%)	6 (13%)	2 (4.2%)	3 (6.3%)	3 (6.3%)	1 (2.1%)	2.15	0.81	5.69	0.13
Penicillins/polymyxins	24 (16%)	2 (1.4%)	3 (2.1%)	22 (15%)	1 (0.7%)	17 (17%)	1 (1%)	2 (2%)	15 (15%)	1 (1%)	7 (15%)	1 (2.1%)	1 (2.1%)	7 (15%)		1.23	0.47	3.20	0.67
Polypeptides	1 (0.7%)			1 (0.7%)							1 (2.1%)			1 (2.1%)					
Quinolones and Fluoroquinolones	7 (4.8%)	7 (4.8%)	1 (0.7%)	1 (0.7%)		4 (4.1%)	4 (4.1%)	1 (1%)			3 (6.3%)	3 (6.3%)		1 (2.1%)		0.64	0.14	2.97	0.57
Category II ^a^																			
Aminoglycosides	5 (3.4%)	5 (3.4%)				2 (2%)	2 (2%)				3 (6.3%)	3 (6.3%)				0.31	0.05	1.94	0.21
Cephalosporins (1^st^ gen)	3 (2.1%)			3 (2.1%)							3 (6.3%)			3 (6.3%)					
Lincosamides	1 (0.7%)			1 (0.7%)							1 (2.1%)			1 (2.1%)					
Macrolides	81 (56%)	61 (42%)	43 (30%)	37 (25 %)	35 (24%)	60 (61%)	44 (45%)	32 (33%)	33 (34%)	32 (33%)	21 (44%)	17 (35%)	11 (23%)	4 (8.3%)	14 (29%)	2.03	1.01	4.09	0.05
Penicillins	74 (51%)	44 (30%)	20 (14%)	61 (42%)	25 (17%)	42 (43%)	21 (21%)	13 (13%)	33 (34%)	17 (17%)	32 (67%)	23 (48%)	7 (15%)	28 (58%)	8 (17%)	0.38	0.18	0.77	0.01
Sulfonamides with trimethoprim (injectable)	54 (37%)	46 (32%)	4 (2.7%)	14 (9.6%)	2 (1.4%)	32 (33%)	28 (29%)	2 (2%)	8 (8.2%)		22 (46%)	18 (38%)	2 (4.2%)	6 (13%)	2 (4.2%)	0.57	0.28	1.16	0.12
Category III ^a^																			
Amphenicols	107 (73%)	99 (68%)	58 (40%)	24 (16%)	9 (6.2%)	82 (84%)	76 (78%)	47 (48%)	17 (17%)	6 (6.1%)	25 (52%)	23 (48%)	11 (23%)	7 (15%)	3 (6.3%)	4.72	2.16	10.3	0.001
Sulfonamides	57 (39%)	56 (38%)	1 (0.7%)	2 (1.4%)		39 (40%)	38 (39%)	1 (1%)	1 (1%)		18 (38%)	18 (38%)		1 (2.1%)		1.10	0.54	2.24	0.79
Tetracyclines	118 (81%)	77 (53%)	57 (39%)	104 71%)	62 (43%)	83 (85%)	55 (56%)	40 (41%)	73 (75%)	48 (49%)	35 (73%)	22 (46%)	17 (35%)	31 (65%)	14 (29%)	2.06	0.87	4.77	0.09
Total Herds Reporting Use	145 (99%)	138 (95%)	96 (66%)	138 (95%)	95 (65%)	98 (100%)	96 (98%)	70 (71%)	94 (96%)	73 (75%)	47 (98%)	42 (88%)	26 (54%)	44 (92%)	22 (46%)				

^a^ Health Canada 2009, underlined product defines the category.

**Table A11:** Summary of antimicrobials used by antimicrobial class and frequency of use in nursing calves from July 2019 to June 2020 in 146 cow–calf herds.

Antimicrobial Class	Herds Reporting AMU (%)	<5%	6–30%	31–70%	71–100%	% Treated Not Reported
Nursing calves						
Category I ^b^						
Cephalosporins (3rd generation)	19 (13%)	17 (12%)	4 (2.7%)	1 (0.7%)		2 (1.4%)
Penicillins/polymyxins	2 (1.4%)	1 (0.7%)	1 (0.7%)			
Polypeptides						
Quinolones and fluoroquinolones	7 (4.8%)	5 (3.4%)	3 (2.1%)			1 (0.7%)
Category II ^b^						
Aminoglycosides	5 (3.4%)	2 (1.4%)	3 (2.1%)			
Cephalosporins (1st generation)						
Lincosamides						
Macrolides	61 (42%)	47 (32%)	11 (7.5%)	4 (2.7%)	1 (0.7%)	4 (2.7%)
Penicillins	44 (30%)	32 (22%)	10 (6.8%)	1 (0.7%)	5 (3.4%)	2 (1.4%)
Sulfonamides with trimethoprim (injectable)	46 (32%)	32 (22%)	13 (8.9%)	2 (1.4%)		2 (1.4%)
Category III ^b^						
Amphenicols	99 (68%)	86 (59%)	26 (18%)			4 (2.7%)
Sulfonamides (oral)	56 (38%)	33 (23%)	17 (12%)	3 (2.1%)		4 (2.7%)
Tetracyclines	77 (52.7%)	65 (45%)	11 (7.5%)		6 (4.1%)	4 (2.7%)
Total herds reporting use ^a^	138 (95%)	129 (88%)	59 (40%)	7 (4.8%)	12 (8.2%)	15 (10%)

^a^ Rows and columns might not add up to the number of unique herds as herds can use an antimicrobial class for different reasons at more than one frequency and more than one antimicrobial class at the same frequency. ^b^ Health Canada 2009, underlined product defines the category.

**Table A12:** Summary of antimicrobials used by antimicrobial class and frequency of use in weaned calves from July 2019 to June 2020 in 146 cow–calf herds.

Antimicrobial Class	Herds Reporting AMU (%)	<5%	6–30%	31–70%	71–100%	% Treated Not Reported
Weaned Calves						
Category I ^b^						
Cephalosporins (3rd generation)	7 (4.8%)	7 (4.8%)	1 (0.7%)			
Penicillins/polymyxins	3 (2.1%)	3 (2.1%)				
Polypeptides						
Quinolones and fluoroquinolones	1 (0.7%)	1 (0.7%)				
Category II ^b^						
Aminoglycosides						
Cephalosporins (1st generation)						
Lincosamides						
Macrolides	43 (30%)	38 (26%)	4 (2.7%)		2 (1.4%)	1 (0.7%)
Penicillins	20 (14%)	17 (12%)	4 (2.7%)			
Sulfonamides with trimethoprim (injectable)	4 (2.7%)	3 (2.1%)	1 (0.7%)			
Category III ^b^						
Amphenicols	58 (40%)	46 (32%)	8 (5.5%)	1 (0.7%)	1 (0.7%)	2 (1.4%)
Sulfonamides	1 (0.7%)	1 (0.7%)				
Tetracyclines	57 (39%)	49 (34%)	9 (6.2%)	1 (0.7%)	4 (2.7%)	3 (2.1%)
Total herds reporting use ^a^	96 (66%)	88 (61%)	23 (16%)	2 (1.4%)	5 (3.4%)	5 (3.4%)

^a^ Rows and columns might not add up to the number of unique herds as herds can use an antimicrobial class for different reasons at more than one frequency and more than one antimicrobial class at the same frequency. ^b^ Health Canada 2009, underlined product defines the category.

**Table A13:** Summary of antimicrobials used by antimicrobial class and frequency of use in cows from July 2019 to June 2020 in 146 cow–calf herds.

Antimicrobial Class	Total Herds Reporting AMU (%)	<5%	6–30%	31–70%	71–100%	% Treated Not Reported
Cows						
Category I ^b^						
Cephalosporins (3rd generation)	13 (8.9%)	12 (8.2%)	1 (0.7%)			1 (0.7%)
Penicillins/polymyxins	22 (15%)	19 (13%)	3 (2.1%)			
Polypeptides	1 (0.7%)	1 (0.7%)				
Quinolones and fluoroquinolones	1 (0.7%)	1 (0.7%)				
Category II ^b^						
Aminoglycosides						
Cephalosporins (1st generation)	3 (2.1%)	2 (1.4%)	1 (0.7%)			
Lincosamides	1 (0.7%)	1 (0.7%)				
Macrolides	37 (25%)	37 (25%)	1 (0.7%)			3 (2.1%)
Penicillins	61 (42%)	57 (39%)	5 (3.4%)			3 (2.1%)
Sulfonamides with trimethoprim (injectable)	15 (10%)	12 (8.2%)				3 (2.1%)
Category III ^b^						
Amphenicols	24 (16.4%)	22 (15%)	2 (1.4%)			
Sulfonamides (uterine)	2 (1.4%)	1 (0.7%)				1 (0.7%)
Tetracyclines	104 (71%)	94 (64%)	19 (13%)			6 (4.1%)
Total herds reporting use ^a^	138 (95%)	133 (91%)	26 (18%)	0 (0%)	0 (0%)	13 (8.9%)

^a^ Rows and columns might not add up to the number of unique herds as herds can use an antimicrobial class for different reasons at more than one frequency and more than one antimicrobial class at the same frequency. ^b^ Health Canada 2009, underlined product defines the category.

**Table A14:** Summary of antimicrobials used by antimicrobial class and frequency of use in bulls from July 2019 to June 2020 in 146 cow–calf herds.

Antimicrobial Class	Herds Reporting AMU (%)	<5%	6–30%	31–70%	71–100%	% Treated Not Reported
Bulls						
Category I ^b^						
Cephalosporins (3rd generation)	4 (2.7%)	3 (2.1%)	1 (0.7%)			
Penicillins/polymyxins	1 (0.7%)	1 (0.7%)				
Polypeptides						
Category II ^b^						
Aminoglycosides						
Cephalosporins (1st generation)						
Lincosamides						
Macrolides	35 (24%)	29 (20%)	2 (1.4%)	1 (0.7%)		4 (2.7%)
Penicillins	25 (17%)	17 (12%)	4 (2.7%)	2 (1.4%)		2 (1.4%)
Sulfonamides with trimethoprim (injectable)	2 (1.4%)		1 (0.7%)			1 (0.7%)
Category III ^b^						
Amphenicols	9 (6.2%)	6 (4.1%)	1 (0.7%)			2 (1.4%)
Sulfonamides						
Tetracyclines	62 (43%)	48 (33%)	11 (7.5%)	1 (0.7%)		4 (2.7%)
Total herds reporting use ^a^	95 (65%)	77 (53%)	16 (11%)	4 (2.7%)	0 (0%)	9 (6.2%)

^a^ Rows and columns might not add up to the number of unique herds as herds can use an antimicrobial class for different reasons at more than one frequency and more than one antimicrobial class at the same frequency. ^b^ Health Canada 2009, underlined product defines the category.

**Table A15:** Summary of antimicrobials used by antimicrobial class and maximum frequency of use at least once in nursing calves or cows for at least one reason from July 2019 to June 2020 in 146 cow–calf herds.

Antimicrobial Class	Herds Reporting AMU (%)	<5%	6–30%	31–70%	71–100%	% Not Reported
Nursing Calves and Cows						
Category I ^b^						
Cephalosporins (3rd generation)	27 (19%)	22 (15%)	3 (2.1%)	1 (0.7%)		3 (2.1%)
Penicillins/polymyxins	23 (16%)	19 (13%)	4 (2.7%)			
Polypeptides	1 (0.7%)	1 (0.7%)				
Quinolones and fluoroquinolones	7 (4.8%)	4 (2.7%)	3 (2.1%)			1 (0.7%)
Category II ^b^						
Aminoglycosides	5 (3.4%)	2 (1.4%)	3 (2.1%)			
Cephalosporins (1st generation)	3 (2.1%)	2 (1.4%)	1 (0.7%)			
Lincosamides	1 (0.7%)	1 (0.7%)				
Macrolides	69 (47%)	50 (34%)	12 (8.2%)	4 (2.7%)	1 (0.7%)	7 (4.8%)
Penicillins	66 (45%)	48 (33%)	11 (7.5%)	1 (0.7%)	5 (3.4%)	4 (2.7%)
Sulfonamides with trimethoprim (injectable)	53 (36%)	37 (25%)	13 (8.9%)	2 (1.4%)		5 (3.4%)
Category III ^b^						
Amphenicols	104 (71%)	75 (51%)	27 (19%)			4 (2.7%)
Sulfonamides	57 (39%)	33 (23%)	17 (12%)	3 (2.1%)		5 (3.4%)
Tetracyclines	115 (79%)	81 (56%)	25 (17%)		6 (4.1%)	9 (6.2%)
Total herds reporting use ^a^	144 (99%)	143 (98%)	70 (48%)	7 (4.8%)	12 (8.2%)	22 (15%)

^a^ Rows and columns might not add up to the number of unique herds as herds can use an antimicrobial class for different reasons at more than one frequency and more than one antimicrobial class at the same frequency. ^b^ Health Canada 2009, underlined product defines the category.

**Table A16:** Unconditional associations for herds reporting treatment of more than 5% of the herd for specific diseases (n *=* 146).

Potential Risk Factors for the Three Most Frequent Reasons for AMU	Odds Ratio	95% CI		*p*-Value
		Lower	Upper	
Preweaning Calf Respiratory Disease				
Herd located in Western Canada: Eastern Canada	0.68	0.29	1.55	0.35
Herd size >300	0.91	0.34	2.47	0.85
Purchased animals during calving season > 2	1.31	0.56	2.06	0.53
Background calves	0.82	0.34	1.87	0.64
At least 1 decision maker < 30	0.79	0.25	2.51	0.68
Maintain individual animal records	0.44	0.18	1.08	0.07
Maintain herd level records	0.56	0.25	1.26	0.16
Start of calving season				0.04
December–February vs. March–May	2.9	1.2	7.0	0.02
June–November vs. March–May	3.0	0.66	13	0.16
Calved on pasture vs. confinement	0.58	0.25	1.32	0.20
Use community pasture	1.55	0.61	3.95	0.36
Use synchronization program	2.10	0.91	4.81	0.08
Use AI	2.85	1.25	6.50	0.01
Preweaning Calf Diarrhea				
Herd located in Western Canada: Eastern Canada	0.77	0.34	1.74	0.53
Herd size > 300	0.63	0.22	1.79	0.38
Purchased animals during calving season > 2	0.89	0.40	1.97	0.77
Background calves	0.94	0.42	2.11	0.87
At least 1 decision maker < 30	0.49	0.13	1.75	0.27
Maintain individual animal records	0.95	0.37	2.47	0.92
Maintain herd level records	0.76	0.34	1.70	0.50
Start of calving season				0.36
December–February vs. March–May	1.8	0.80	4.1	0.15
June–November vs. March–May	1.2	0.22	6.1	0.86
Calved on pasture vs. confinement	0.60	0.27	1.34	0.21
Use community pasture	1.11	0.43	2.88	0.83
Use synchronization program	1.29	0.56	2.96	0.56
Use AI	1.46	0.66	3.21	0.35
Cow Lameness				
Herd located in Western Canada: Eastern Canada	0.57	0.21	1.55	0.27
Herd size > 300	0.43	0.09	1.97	0.27
Purchased animals during year > 4	0.68	0.25	1.85	0.45
Background calves	0.68	0.25	1.84	0.45
At least 1 decision maker < 30	2.35	0.75	7.39	0.14
Maintain individual animal records	1.40	0.38	5.19	0.61
Maintain herd level records	1.21	0.39	3.19	0.83
Start of calving season				0.60
December–February vs. March–May	1.7	0.60	4.6	0.33
June–November vs. March–May	0.99	0.11	8.8	0.99
Calved on pasture vs. confinement	0.68	0.25	1.86	0.45
Use community pasture	2.17	0.74	6.35	0.16
Use synchronization program	1.57	0.56	4.35	0.39
Use AI	1.61	0.60	4.34	0.35
