# Supplementary material for: Antimicrobial Use in Canadian Cow–Calf Herds
Source: Vet Sci. 2023 May 20;10(5):366. doi: 10.3390/vetsci10050366 (PMC10223781; doi:10.3390/vetsci10050366)
Supplement: Supplementary file 1 [file vetsci-10-00366-s001.zip › vetsci-2356764-supplementary.pdf]

Antibiotic Use in
Canadian Cow-Calf Herds

C3SN
CANADIAN COW – CALF SURVEILLANCE NETWORK

This survey is part of the research for the Canadian Cow-Calf Surveillance Network that is being conducted by researchers from the University of Saskatchewan and our collaborators at the University of Guelph, Université de Montréal and University of Calgary.

Your participation is voluntary. All of your responses will be kept confidential.
Return of the survey by mail will indicate your consent to participate
and have your deidentified responses summarized in the final report.

- **Please do not leave any blank spaces**, answer each question in the survey as best as you can. Either cross out the answer or write:
  - ❖ **'0's'** - there were no events in this category
  - ❖ or **'Not Applicable' (N/A)** - does not apply to my farm
  - ❖ or **'Don't Know'** - we do not have records specific to this question
- Please send back each page of the survey.
- Please return the survey in the stamped envelope provided

Please enter your full name: ______________________________

If you have any questions regarding the questionnaire, feel free to call:

Sharlene April at the Western College of Veterinary Medicine
Phone: (306) 966-7870
or e-mail c3sn@usask.ca

or

Dr. Cheryl Waldner at the Western College of Veterinary Medicine
Phone: (306) 966-7168
or e-mail cheryl.waldner@usask.ca

Check out our website: https://research-groups.usask.ca/c3sn
Follow us on Twitter: @TheC3SN

The intent of this questionnaire is to understand antibiotic use in Canadian cow-calf herds between July 1, 2019 and June 30, 2020.

For the purpose of this survey, the term antibiotic
will be considered equivalent to the term antimicrobial.

Antibiotics *or* ***antimicrobials*** *are* ***used in livestock production***
to treat or control bacterial infections*.*

They include injectable medications, boluses, and feed and water additives such as tetracyclines, macrolides, sulfonamides and ionophores/coccidiostats.
They do not include vaccines, treatments for lice, dewormers
or nutritional supplements.

Antibiotics or antimicrobials might be used to ***treat animals that are sick and we are trying to make them better****. Antibiotics or antimicrobials might also be used in* ***animals that do not appear to be sick****, but where they are* ***likely to become sick if nothing is done to prevent or control the disease****.*

PART A.

Note: This page contains important examples and definitions. Please read and refer to it as you answer the survey questions.

Please fill out the following tables regarding the **antibiotics given to your cow-calf herd.** This includes all antibiotics which are:

- ✓ injected into muscle (IM), underneath the skin (SC) or into a blood vessel (IV)
- ✓ inserted directly into the udder (IMM)
- ✓ inserted directly into the uterus (IU)
- ✓ fed to the cattle, added to the water, or given by mouth (PO)
- ✓ applied to the surface of the eyes or skin (topical)

Please indicate:

- ✓ which antibiotics were used to treat cattle for each reason listed in the tables
- ✓ the percentage of animals treated in each age class since July 1, 2019
- ✓ the number of doses you would typically use to treat an animal for that reason with that drug
- ✓ the typical dose per animal
- ✓ the typical route of administration

If you have not treated any animals in a specific age class with antibiotics for a particular reason since July 1, 2019, simply check the appropriate box in the first column (*Please see the example below for Respiratory Disease in breeding females).

You can refer to the Antibiotic Handbook for a list of currently available antibiotics to help you find the names of the products used in your herd.

Fill out one line per antibiotic used for each reason.

Please refer to this example to answer the questions:

Breeding Females:

Reason for Antibiotic Use	*Which Antibiotic(s) Did You Use?*	*Percentage of Animals Treated since July 1st 2019* **(select only 1 category)**	*Typical Number of Doses Used/ Dose per Animal/ Route?*
Mammary/Udder Disease e.g. mastitis<br>☐ *No animals treated for this condition since July 1, 2019*	1. ***Excenel***	☐ *<5%* ☐ *31-70 %*<br>☒ *6-30%* ☐ *71-100%*	***3 doses/14 mL per dose SC***
	2 ***Special Formula***	☒ *<5%* ☐ *31-70 %*<br>☐ *6-30%* ☐ *71-100%*	***2 doses/ 1 tube per day IMM***
	3. ---	☐ *<5%* ☐ *31-70 %*<br>☐ *6-30%* ☐ *71-100%*	
Respiratory Disease e.g. pneumonia<br>☒ *No animals treated for this condition since July 1, 2019*	1. ---	☐ *<5%* ☐ *31-70 %*<br>☐ *6-30%* ☐ *71-100%*	
	2. ---	☐ *<5%* ☐ *31-70 %*<br>☐ *6-30%* ☐ *71-100%*	
	3. ---	☐ *<5%* ☐ *31-70 %*<br>☐ *6-30%* ☐ *71-100%*	
Lameness e.g. foot rot<br>☐ *No animals treated for this condition since July 1, 2019*	1. ***Nuflor***	☒ *<5%* ☐ *31-70 %*<br>☐ *6-30%* ☐ *71-100%*	***Day 1 & 3 / 40 ml per day IM***
	2.	☐ *<5%* ☐ *31-70 %*<br>☐ *6-30%* ☐ *71-100%*	
	3.	☐ *<5%* ☐ *31-70 %*<br>☐ *6-30%* ☐ *71-100%*	

1. Preweaned Calves: *(Calves born in 2019 and treated from July 1st to weaning, as well as calves born in 2020 and treated from birth to June 30th)*

What month did you start calving in 2019? ____________________

What month did you start calving in 2020? ____________________

**Reason for Antibiotic Use**	**Which Antibiotic(s) Did You Use?**	**Percentage of Animals Treated** since July 1st 2019 (select only 1 category)	**Typical Number of Doses Used/ Dose per Animal/ Route?**
**Umbilical infection/Navel ill**<br><br>☐ *No preweaned calves treated for this reason since July 1, 2019*	1.	☐ *<5%* ☐ *31-70%*<br>☐ *6-30%* ☐ *71-100%*	
	2	☐ *<5%* ☐ *31-70%*<br>☐ *6-30%* ☐ *71-100%*	
	3.	☐ *<5%* ☐ *31-70%*<br>☐ *6-30%* ☐ *71-100%*	
	4.	☐ *<5%* ☐ *31-70%*<br>☐ *6-30%* ☐ *71-100%*	
**Respiratory Disease *e.g. pneumonia / shipping fever***<br><br>☐ *No preweaned calves treated for this reason since July 1, 2019*	1.	☐ *<5%* ☐ *31-70%*<br>☐ *6-30%* ☐ *71-100%*	
	2	☐ *<5%* ☐ *31-70%*<br>☐ *6-30%* ☐ *71-100%*	
	3.	☐ *<5%* ☐ *31-70%*<br>☐ *6-30%* ☐ *71-100%*	
	4.	☐ *<5%* ☐ *31-70%*<br>☐ *6-30%* ☐ *71-100%*	
**Diarrhea / Bloat**<br><br>***e.g. scours / coccidiosis***<br><br>☐ *No preweaned calves treated for this reason since July 1, 2019*	1.	☐ *<5%* ☐ *31-70%*<br>☐ *6-30%* ☐ *71-100%*	
	2	☐ *<5%* ☐ *31-70%*<br>☐ *6-30%* ☐ *71-100%*	
	3.	☐ *<5%* ☐ *31-70%*<br>☐ *6-30%* ☐ *71-100%*	
	4.	☐ *<5%* ☐ *31-70%*<br>☐ *6-30%* ☐ *71-100%*	
**Arthritis / Lameness *e.g. joint ill / foot rot***<br><br>☐ *No preweaned calves treated for this reason since July 1, 2019*	1.	☐ *<5%* ☐ *31-70%*<br>☐ *6-30%* ☐ *71-100%*	
	2	☐ *<5%* ☐ *31-70%*<br>☐ *6-30%* ☐ *71-100%*	
	3.	☐ *<5%* ☐ *31-70%*<br>☐ *6-30%* ☐ *71-100%*	
	4.	☐ *<5%* ☐ *31-70%*<br>☐ *6-30%* ☐ *71-100%*	
**Disease Prevention**<br>*For what reason(s)?*[1]<br>i.____________________	1.	☐ *<5%* ☐ *31-70%*<br>☐ *6-30%* ☐ *71-100%*	
	2	☐ *<5%* ☐ *31-70%*<br>☐ *6-30%* ☐ *71-100%*	
ii.____________________<br>☐ *No preweaned calves given antibiotics for prevention since July 1, 2019*	1.	☐ *<5%* ☐ *31-70%*<br>☐ *6-30%* ☐ *71-100%*	
	2.	☐ *<5%* ☐ *31-70%*<br>☐ *6-30%* ☐ *71-100%*	
**Other reason(s) for use?** **(please specify)**<br>i. ____________________	1.	☐ *<5%* ☐ *31-70%*<br>☐ *6-30%* ☐ *71-100%*	
	2	☐ *<5%* ☐ *31-70%*<br>☐ *6-30%* ☐ *71-100%*	
ii. ____________________<br>☐ *No preweaned calves given antibiotics for other reason(s) since July 1, 2019*	1.	☐ *<5%* ☐ *31-70%*<br>☐ *6-30%* ☐ *71-100%*	
	2.	☐ *<5%* ☐ *31-70%*<br>☐ *6-30%* ☐ *71-100%*	

[1] *Previously reported examples: antibiotics for the prevention of navel ill, scours, or pneumonia*

2. Weaned Calves (*Post-weaning for calves born in 2019 e.g. 2019-born replacement heifers, backgrounders or weaned calves pre-shipping*)

What month did you wean in 2019? ________________

What % of weaned calves did you <u>sell</u> at weaning in 2019? ______________

Reason for Antibiotic Use	Which Antibiotic(s) Did You Use?	Percentage of Animals Treated since July 1st 2019 (select only 1 category)	Typical Number of Doses Used/ Dose per Animal/ Route?
**Eye Disease** ***e.g. pink eye*** ☐ *No post-weaned calves treated for this reason since July 1, 2019*	1.	☐ *<5%* ☐ *31-70 %* ☐ *6-30%* ☐ *71-100%*	
	2	☐ *<5%* ☐ *31-70 %* ☐ *6-30%* ☐ *71-100%*	
	3.	☐ *<5%* ☐ *31-70 %* ☐ *6-30%* ☐ *71-100%*	
	4.	☐ *<5%* ☐ *31-70 %* ☐ *6-30%* ☐ *71-100%*	
**Respiratory Disease** ***e.g. pneumonia / shipping fever*** ☐ *No post-weaned calves treated for this reason since July 1, 2019*	1.	☐ *<5%* ☐ *31-70 %* ☐ *6-30%* ☐ *71-100%*	
	2	☐ *<5%* ☐ *31-70 %* ☐ *6-30%* ☐ *71-100%*	
	3.	☐ *<5%* ☐ *31-70 %* ☐ *6-30%* ☐ *71-100%*	
	4.	☐ *<5%* ☐ *31-70 %* ☐ *6-30%* ☐ *71-100%*	
**Diarrhea / Bloat** ***e.g. coccidiosis*** ☐ *No post-weaned calves treated for this reason since July 1, 2019*	1.	☐ *<5%* ☐ *31-70 %* ☐ *6-30%* ☐ *71-100%*	
	2	☐ *<5%* ☐ *31-70 %* ☐ *6-30%* ☐ *71-100%*	
	3.	☐ *<5%* ☐ *31-70 %* ☐ *6-30%* ☐ *71-100%*	
	4.	☐ *<5%* ☐ *31-70 %* ☐ *6-30%* ☐ *71-100%*	
**Lameness** ***e.g. foot rot / arthritis*** ☐ *No post-weaned calves treated for this reason since July 1, 2019*	1.	☐ *<5%* ☐ *31-70 %* ☐ *6-30%* ☐ *71-100%*	
	2	☐ *<5%* ☐ *31-70 %* ☐ *6-30%* ☐ *71-100%*	
	3.	☐ *<5%* ☐ *31-70 %* ☐ *6-30%* ☐ *71-100%*	
	4.	☐ *<5%* ☐ *31-70 %* ☐ *6-30%* ☐ *71-100%*	
**Disease Prevention** ***e.g. pneumonia / liver abscesses*** *Which disease(s)?* i.__________________ ii.__________________ ☐ *No post-weaned calves given antibiotics for prevention since July 1, 2019*	1.	☐ *<5%* ☐ *31-70 %* ☐ *6-30%* ☐ *71-100%*	
	2	☐ *<5%* ☐ *31-70 %* ☐ *6-30%* ☐ *71-100%*	
	1.	☐ *<5%* ☐ *31-70 %* ☐ *6-30%* ☐ *71-100%*	
	2.	☐ *<5%* ☐ *31-70 %* ☐ *6-30%* ☐ *71-100%*	
**Other reason(s) for use? (please specify)** i.__________________ ii.__________________	1.	☐ *<5%* ☐ *31-70 %* ☐ *6-30%* ☐ *71-100%*	
	2	☐ *<5%* ☐ *31-70 %* ☐ *6-30%* ☐ *71-100%*	
	1.	☐ *<5%* ☐ *31-70 %* ☐ *6-30%* ☐ *71-100%*	
	2.	☐ *<5%* ☐ *31-70 %* ☐ *6-30%* ☐ *71-100%*	

3. Breeding Females: *(Treated between July 1st 2019 and June 30th 2020)*

**Reason for Antibiotic Use in Cows/Heifers**	**Which Antibiotic(s) Did You Use?**	**Percentage of Animals Treated since July 1st 2019 (select only 1 category)**	**Typical Number of Doses Used/ Dose per Animal/ Route?**
**Eye Disease** ***e.g. pink eye*** ☐ *No cows/heifers treated for this reason since July 1, 2019*	1.	☐ *<5%* ☐ *31-70 %* ☐ *6-30%* ☐ *71-100%*	
	2	☐ *<5%* ☐ *31-70 %* ☐ *6-30%* ☐ *71-100%*	
	3.	☐ *<5%* ☐ *31-70 %* ☐ *6-30%* ☐ *71-100%*	
	4.	☐ *<5%* ☐ *31-70 %* ☐ *6-30%* ☐ *71-100%*	
**Respiratory Disease** ***e.g. pneumonia / shipping fever*** ☐ *No cows/heifers treated for this reason since July 1, 2019*	1.	☐ *<5%* ☐ *31-70 %* ☐ *6-30%* ☐ *71-100%*	
	2.	☐ *<5%* ☐ *31-70 %* ☐ *6-30%* ☐ *71-100%*	
	3.	☐ *<5%* ☐ *31-70 %* ☐ *6-30%* ☐ *71-100%*	
	4.	☐ *<5%* ☐ *31-70 %* ☐ *6-30%* ☐ *71-100%*	
**Mammary / Udder Disease** ***e.g. mastitis*** ☐ *No cows/heifers treated for this reason since July 1, 2019*	1.	☐ *<5%* ☐ *31-70 %* ☐ *6-30%* ☐ *71-100%*	
	2.	☐ *<5%* ☐ *31-70 %* ☐ *6-30%* ☐ *71-100%*	
	3.	☐ *<5%* ☐ *31-70 %* ☐ *6-30%* ☐ *71-100%*	
	4.	☐ *<5%* ☐ *31-70 %* ☐ *6-30%* ☐ *71-100%*	
**Lameness** ***e.g. foot rot / arthritis*** ☐ *No cows/heifers treated for this reason since July 1, 2019*	1.	☐ *<5%* ☐ *31-70 %* ☐ *6-30%* ☐ *71-100%*	
	2.	☐ *<5%* ☐ *31-70 %* ☐ *6-30%* ☐ *71-100%*	
	3.	☐ *<5%* ☐ *31-70 %* ☐ *6-30%* ☐ *71-100%*	
	4.	☐ *<5%* ☐ *31-70 %* ☐ *6-30%* ☐ *71-100%*	
**Reproductive Tract Disease** ***e.g. metritis / uterine infection / retained placenta*** ☐ *No cows/heifers treated for this reason since July 1, 2019*	1.	☐ *<5%* ☐ *31-70 %* ☐ *6-30%* ☐ *71-100%*	
	2.	☐ *<5%* ☐ *31-70 %* ☐ *6-30%* ☐ *71-100%*	
	3.	☐ *<5%* ☐ *31-70 %* ☐ *6-30%* ☐ *71-100%*	
	4.	☐ *<5%* ☐ *31-70 %* ☐ *6-30%* ☐ *71-100%*	
**Disease Prevention (please specify)** i.___________________ ii.___________________ ***e.g. coccidiosis / bloat*** ☐ *No cows/heifers given antibiotics for prevention since July 1, 2019*	1.	☐ *<5%* ☐ *31-70 %* ☐ *6-30%* ☐ *71-100%*	
	2.	☐ *<5%* ☐ *31-70 %* ☐ *6-30%* ☐ *71-100%*	
	3.	☐ *<5%* ☐ *31-70 %* ☐ *6-30%* ☐ *71-100%*	
	4.	☐ *<5%* ☐ *31-70 %* ☐ *6-30%* ☐ *71-100%*	
**Other reason(s) for use? (please specify)** i.___________________ ii.___________________	1.	☐ *<5%* ☐ *31-70 %* ☐ *6-30%* ☐ *71-100%*	
	2.	☐ *<5%* ☐ *31-70 %* ☐ *6-30%* ☐ *71-100%*	
	1.	☐ *<5%* ☐ *31-70 %* ☐ *6-30%* ☐ *71-100%*	

4. Bulls: *(Treated between July 1st 2019 and June 30th 2020)*

Reason for Antibiotic Use	Which Antibiotic(s) Did You Use?	Percentage of Animals Treated since July 1st 2019 (select only 1 category)	Typical Number of Doses Used/ Dose per Animal/ Route?
**Eye Disease**<br>***e.g. pink eye***<br>☐ *No bulls treated for this reason since July 1, 2019*	1.	☐ *<5%* ☐ *31-70 %*<br>☐ *6-30%* ☐ *71-100%*	
	2.	☐ *<5%* ☐ *31-70 %*<br>☐ *6-30%* ☐ *71-100%*	
	3.	☐ *<5%* ☐ *31-70 %*<br>☐ *6-30%* ☐ *71-100%*	
	4.	☐ *<5%* ☐ *31-70 %*<br>☐ *6-30%* ☐ *71-100%*	
**Respiratory Disease**<br>***e.g. pneumonia***<br>☐ *No bulls treated for this reason since July 1, 2019*	1.	☐ *<5%* ☐ *31-70 %*<br>☐ *6-30%* ☐ *71-100%*	
	2.	☐ *<5%* ☐ *31-70 %*<br>☐ *6-30%* ☐ *71-100%*	
	3.	☐ *<5%* ☐ *31-70 %*<br>☐ *6-30%* ☐ *71-100%*	
	4.	☐ *<5%* ☐ *31-70 %*<br>☐ *6-30%* ☐ *71-100%*	
**Lameness**<br>***e.g. foot rot / arthritis***<br>☐ *No bulls treated for this reason since July 1, 2019*	1.	☐ *<5%* ☐ *31-70 %*<br>☐ *6-30%* ☐ *71-100%*	
	2.	☐ *<5%* ☐ *31-70 %*<br>☐ *6-30%* ☐ *71-100%*	
	3.	☐ *<5%* ☐ *31-70 %*<br>☐ *6-30%* ☐ *71-100%*	
	4.	☐ *<5%* ☐ *31-70 %*<br>☐ *6-30%* ☐ *71-100%*	
**Reproductive Tract Disease**<br>***e.g. penis / prepuce injury***<br>☐ *No bulls treated for this reason since July 1, 2019*	1.	☐ *<5%* ☐ *31-70 %*<br>☐ *6-30%* ☐ *71-100%*	
	2.	☐ *<5%* ☐ *31-70 %*<br>☐ *6-30%* ☐ *71-100%*	
	3.	☐ *<5%* ☐ *31-70 %*<br>☐ *6-30%* ☐ *71-100%*	
	4.	☐ *<5%* ☐ *31-70 %*<br>☐ *6-30%* ☐ *71-100%*	
**Disease Prevention**<br>**(please describe)**<br>i. ____________________<br>ii. ____________________<br>☐ *No bulls given antibiotics for prevention since July 1, 2019*	1.	☐ *<5%* ☐ *31-70 %*<br>☐ *6-30%* ☐ *71-100%*	
	2	☐ *<5%* ☐ *31-70 %*<br>☐ *6-30%* ☐ *71-100%*	
	1.	☐ *<5%* ☐ *31-70 %*<br>☐ *6-30%* ☐ *71-100%*	
	2	☐ *<5%* ☐ *31-70 %*<br>☐ *6-30%* ☐ *71-100%*	
**Other reason(s) for use? (please describe)**<br>i.____________________<br>ii.____________________	1.	☐ *<5%* ☐ *31-70 %*<br>☐ *6-30%* ☐ *71-100%*	
	2.	☐ *<5%* ☐ *31-70 %*<br>☐ *6-30%* ☐ *71-100%*	
	1.	☐ *<5%* ☐ *31-70 %*<br>☐ *6-30%* ☐ *71-100%*	
	2.	☐ *<5%* ☐ *31-70 %*<br>☐ *6-30%* ☐ *71-100%*	

PART B.

Please circle only one of the following responses to the following questions:

1. Over the last 5 years, I have noticed the effectiveness of antibiotics for treating RESPIRATORY disease (e.g. pneumonia in a preweaned calf or shipping fever for post weaned calves) on my farm has:

   INCREASED STAYED THE SAME DECREASED NO OPINION

2. Over the last 5 years, I have noticed the effectiveness of antibiotics for treating GASTROINTESTINAL disease (e.g. calf scours in a 3-day old calf) on my farm has:

   INCREASED STAYED THE SAME DECREASED NO OPINION

3. Over the last 5 years, I have noticed the effectiveness of antibiotics for treating LAMENESS (e.g. foot rot in mature cows) on my farm has:

   INCREASED STAYED THE SAME DECREASED NO OPINION

4. Reports of antibiotic resistance in cattle and the potential loss of treatment effectiveness from antibiotic resistance in my herd has limited the choice of drugs used to treat sick animals in my herd in the past 5 years:

   AGREE NEUTRAL DISAGREE NO OPINION

5. The potential for antibiotic use in cattle to impact antibiotic resistance in human medicine has limited the use of Category 1 drugs (*e.g. Excenel, Excede, Baytril*) to treat sick animals in my herd in the past 5 years:

   AGREE NEUTRAL DISAGREE NO OPINION

For questions 6 to 9, please place an X beside up to 3 options that best represent your management practices. Only X the options you actually use. Leave the rest blank.

Please refer to this example to answer the following questions:
If your first antibiotic treatment failed in treating GASTROINTESTINAL disease, what was your next course of action in the last year?

__ *Provide no further treatment*
__ *Treat with a different antibiotic*
_X_ *Treat with a different drug/product that is not an antibiotic (Please specify):* Oral electrolytes
_X_ *Consult a veterinarian*
__ *Cull or euthanize the animal*
__ *Other (Please specify)* ____________________________

6. If your first antibiotic treatment failed in treating RESPIRATORY disease (e.g. pneumonia in a preweaned calf), what was your next course of action in the last year?

____ Provide no further treatment
____ Treat with a different antibiotic
____ Treat with a different drug/product that is not an antibiotic
(Please specify): ____
____ Cull or euthanize the animal
____ Consult a veterinarian
____ Other (Please specify): ____

7. If your first antibiotic treatment failed in treating GASTROINTESTINAL disease (e.g. scours in a 3-day old calf), what was your next course of action in the last year?

____ Provide no further treatment
____ Treat with a different antibiotic
____ Treat with a different drug/product that is not an antibiotic
(Please specify): ____
____ Cull or euthanize the animal
____ Consult a veterinarian
____ Other (Please specify): ____

8. If your first antibiotic treatment failed in treating LAMENESS (e.g. foot rot in a mature cow), what was your next course of action in the last year?

____ Provide no further treatment
____ Treat with a different antibiotic
____ Treat with a different drug/product that is not an antibiotic
(Please specify): ____
____ Cull or euthanize the animal
____ Consult a veterinarian
____ Other (Please specify): ____

9. What are your main sources of information regarding antibiotics?

____ Your veterinarian
____ Feed or drug company representatives
____ Industry/drug company websites
____ Beef industry meetings/trade shows
____ Friends and neighbors
____ Government publications/ websites
____ Nutritionists
____ Producer publications (e.g. newspapers, magazines)
____ Scientific journals
____ Social media/blogs
____ Other (Please specify): ____

10. Select your <u>top 3 reasons</u> for deciding <u>whether or not to treat</u> a calf/cow/bull with antibiotics when it is sick: *(Place an* **X** *beside your <u>3</u> choices)*

____ Verbal (phone/in-person) recommendations from my veterinarian **for this animal**

____ Written recommendations from my veterinarian specific **for this animal**

____ Written <u>treatment protocol</u> from my veterinarian **for my herd** describing who to treat and when to treat

____ Verbal (phone/in-person) advice from my veterinarian **not specific** to this animal

____ Drug cost versus chance of success?

____ Drug accessibility – i.e. can I buy it when I need it?

____ Withdrawal time

____ Whether more than 1 dose is needed

____ Route of administration (under skin, in muscle, oral (bolus, in feed, in water), etc.)

____ Cost of the disease in my herd (# sick, lost performance (i.e. decreased gain, pregnancy loss), # dead)

____ Severity of the disease / clinical signs – i.e. how sick is the animal?

____ Concern about the animal's well-being

____ Potential for the drug not to work due to antibiotic resistance

____ Other (Please specify): ____

____ **Not applicable** – I do not use antibiotics in my animals

11. Select your <u>top 3 reasons</u> for deciding <u>which antibiotic to use</u> in treating one of your calves/cows/bulls when it is sick: *(Place an* **X** *beside your <u>3</u> choices)*

____ Verbal (phone/in-person) recommendations from my veterinarian **for this animal**

____ Written recommendations from my veterinarian **for this animal**

____ Written <u>treatment protocol</u> from my veterinarian **for my herd** describing who to treat, when to treat, and what product to use

____ Verbal (phone/in-person) advice from my veterinarian **not specific** to this animal

____ Advice from a friend/neighbor/co-worker

____ Drug cost versus chance of success?

____ Drug accessibility – i.e. can I buy it when I need it?

____ Withdrawal time

____ Whether I have to mix the drug before use

____ Whether the drug needs to be refrigerated

____ Potential reactions or side effects of the drug (i.e. safety issues)

____ Whether more than 1 dose is needed

____ Route of administration (under skin, in muscle, oral (bolus, in feed, in water), etc.)

____ Cost of the disease in my herd (# sick, lost performance (i.e. decreased gain, pregnancy loss), # dead)

____ Severity of the disease / clinical signs – i.e. how sick is the animal?

____ Concern about the animal's well-being

____ Potential for antibiotic resistance to result from the use of this drug

____ Potential for the drug not to work due to antibiotic resistance

____ Other (Please specify): ____

____ **Not applicable** – I do not use antibiotics in my animals

12. Select your <u>top 3 reasons</u> for deciding <u>how much antibiotic to use</u> (e.g. how many mL/cc or boluses) **and how many doses you will give to an animal:** *(Place an* **X** *beside your* *<u>3</u>* *choices)*

____ Verbal (phone/in-person) recommendations from my veterinarian **for this animal**

____ Written recommendations from my veterinarian **for this animal**

____ Written <u>treatment protocol</u> from my veterinarian **for my herd** describing who to treat, when to treat, what product to use and how much

____ Verbal (phone/in-person) advice from my veterinarian **not specific** to this animal

____ Advice from a friend/neighbor/co-worker

____ Use the same amount I have always used

____ Based on a visual estimate of the animal's weight

____ Based on a measured weight of the animal (weigh scale)

____ Drug cost

____ Withdrawal time

____ Route of administration (under skin, in muscle, oral (bolus, in feed, in water), etc.)

____ Package label instructions

____ Instructions on the internet

____ Other (Please specify): ____

____ **Not applicable** – I do not use antibiotics in my animals

13. Did you keep <u>individual animal</u> treatment records in 2019/2020?
(e.g. Calf #62 treated for pneumonia with 30 ml Nuflor) (*Check **<u>all</u>** applicable options*)

☐ Yes – handwritten ☐ Yes – computerized ☐ Yes – smartphone ☐ No

14. Did you keep <u>group</u> or <u>herd level</u> treatment records in 2019/2020?
i.e. a written count of animals treated and when (e.g. 8 calves treated for pneumonia with Nuflor) (*Check **<u>all</u>** applicable options*)

☐ Yes – handwritten ☐ Yes – computerized ☐ Yes – smartphone ☐ No

15. Are you aware of initiatives to increase antibiotic stewardship *(ensuring that the beef industry uses the right antibiotics in the right animals at the right dose at the right time)***?**

☐ Yes ☐ No

16. If 'Yes' to question 15, where did you get information about this/these initiative(s)?
*(Please check **<u>all</u>** that apply)*

____ **Not applicable**, I am not aware of any antibiotic stewardship initiatives
____ Veterinarians – verbal recommendation
____ Veterinarians – written treatment protocol
____ Feed or drug company representatives
____ Industry/drug company websites
____ Friends and neighbors
____ Government publications/ websites
____ Nutritionists
____ Beef industry meetings/trade shows
____ Producer publications (newspapers, magazines)
____ Scientific journals
____ Social media/blogs

____ Other (Please specify): ____

17. In your opinion, how important is the issue of antibiotic resistance to the beef industry? *(Circle the best answer)*

VERY IMPORTANT   MODERATELY IMPORTANT   NOT IMPORTANT

In December 2018, there was a change to federal regulations in how livestock producers could purchase medically important antibiotics *(i.e. antimicrobials important to human medicine – excludes ionophores such as Monensin)* **limiting them to being available only through prescriptions from veterinarians.**

You can refer to the Beef Cattle Research Council website for more information:
http://www.beefresearch.ca/blog/prescription-required-for-livestock-cattle-antibiotics-december-2018-repost/

18. Has antibiotic use in your herd been affected by the regulation change that came into effect December 2018? *(Circle the best answer)*

DECREASED   NO CHANGE   INCREASED

Comments:____

____

19. Has your access to antibiotics changed? *(Circle the best answer)*

DECREASED   NO CHANGE   INCREASED

Comments:____

____

20. Has the cost of antibiotic use in your herd changed? *(Circle the best answer)*

DECREASED   NO CHANGE   INCREASED

Comments:____

____

21. How did you first become aware of the need for a prescription to buy antibiotics?
(Please place an **X** *beside the one most appropriate answer)*

____ **Not applicable**, I was not aware of a change in how antibiotics can be purchased for livestock

I first learned about this from:

____ Veterinarians
____ Feed or Drug Company representatives
____ Industry/drug company websites
____ Friends and neighbors
____ Government publications/ websites
____ Nutritionists
____ Beef industry meetings/trade shows
____ Producer publications (e.g. newspapers, magazines)
____ Scientific journals
____ Social media/blogs
____ Webinars

____ I don’t remember

____ Other (Please specify): ____

22. **What percentage of treatments for sick animals do you administer using a remote delivery system for antibiotics** (i.e. tools to help administer injectable antibiotics to cattle that you cannot restrain in a chute system/head gate/squeeze)**?**

*(Please enter the best estimate of the **percentage** of treatments (%) next to all that apply.)*

____ **Not applicable**, we have only administered antibiotics to animals restrained in a chute system, head gate, squeeze or stanchion/tied-stalls

____ Dart gun with medicinal delivery dart

____ Cross bow with medicinal delivery dart

____ Blow gun with medicinal delivery dart

____ Pole syringe

____ Rope and restrain on pasture with traditional syringe

____ In feed or water

____ Other (Please specify): ____

Comments:____

____

23. **Do you have any other comments related to antibiotic use, antibiotic resistance, or the need for antibiotic stewardship in the beef industry?**

____

____

____

____

____

____

____

____

____

24. **Would you be willing to share a copy of your individual animal treatment records with the study in 2021? (*please circle one*)**

YES **MAYBE** **NO**

For example, cow #U42 treated for footrot with Draxxin July 23rd.

- Each herd providing their completed treatment records for the year will receive an additional $300 incentive from the project.
- We will be interested in individual animal and group treatment records collected from January 1, 2021 to December 31, 2021.
- We can provide you with a paper-based or smartphone-based treatment records system for data collection if you would like, or we can use a clear copy, scan or good quality cell phone picture of whatever record system you are using now.
